# Integrative single‐cell transcriptomic analysis deciphers heterogeneous characteristics of gastrointestinal tract cancer

**DOI:** 10.1002/ctm2.70415

**Published:** 2025-08-11

**Authors:** Chuwen Sun, Tong Li, Xin Jin, Zhihui Xiu, Hang Su, Huanming Yang, Ming Liu, Kui Wu

**Affiliations:** ^1^ College of Life Sciences University of Chinese Academy of Sciences Beijing China; ^2^ HIM‐BGI Omics Center Zhejiang Cancer Hospital, Hangzhou Institute of Medicine (HIM), Chinese Academy of Sciences (CAS), BGI Research Hangzhou China; ^3^ Jiangxi Provincial Key Laboratory of Organ Development and Epigenetics, School of Basic Medicine, Jinggangshan University Ji'an China; ^4^ BGI Genomics Harbin Heilongjiang China; ^5^ Guangdong Provincial Key Laboratory of Human Disease Genomics, Shenzhen Key Laboratory of Genomics, BGI Research Shenzhen China; ^6^ Department of General Surgery The Fourth Affiliated Hospital of Harbin Medical University Harbin China

**Keywords:** gastrointestinal tract cancer, intratumoural heterogeneity, metaplasia, single‐cell RNA sequencing, tumour microenvironment

## Abstract

**Background:**

Gastrointestinal tract cancer (GIC), including oesophageal cancer (EC), gastric cancer (GC) and colorectal cancer (CRC), is characterised with high global incidence and mortality rates, with similar tumourigenic processes. However, the common and heterogeneous molecular features among GIC at single‐cell level remain poorly characterised.

**Methods:**

Single‐cell RNA‐seq data of more than one million high‐quality annotated cells from 577 specimens, including 121 ECs, 182 GCs and 254 CRCs, were integrated to systematically decipher the heterogeneous characteristics of GIC. Non‐negative matrix factorisation (NMF) was employed to identify epithelial cell meta‐programs (MPs), and cell–cell communication analysis was conducted to investigate regulatory interactions between the tumour microenvironment (TME) and these MPs. Additionally, cell lineage inference analysis was performed to identify metaplastic signatures in EC and GC.

**Results:**

We identified 24 consensus MPs from epithelial cells and 42 distinct subtypes from non‐epithelial cells thus offering a comprehensive overview of heterogeneous characteristic in GIC. Notably, we observed that EC exhibited unique features, including heightened activity in stress‐related programs and a more exhausted TME, enriched with CD4+ Tregs and CD8+ exhausted T cells. In contrast, epithelial cells in GC displayed increased expression of epithelial–mesenchymal transition (EMT)‐related signatures and an activated immune phenotype, marked by enrichment of NK cells and CD8+ effector T cells. Moreover, samples with metaplastic signatures in GC and EC showed similarities to CRC, including elevated expression of metabolism‐associated signatures and an abundance of CD4+ helper‐like T cells. Finally, we identified the potential regulatory roles of the TME in shaping epithelial cell behaviour.

**Conclusions:**

Our findings provide insights into the common and specific cellular and molecular patterns associated with GIC tumourigenesis and TME remodelling. We also elucidate the similarity between GC/EC with metaplastic signature and CRC, which advancing our understanding of these malignancies.

**Key points:**

- A comprehensive single‐cell atlas of gastrointestinal tract cancer (GIC) was constructed.
- GICs exhibit distinct epithelial features and specific tumour microenvironment (TME) patterns, forming diverse niches.
- GC and EC exhibiting metaplastic features show elevated metabolism‐associated signatures and share similarities with CRC.

## Graphical abstract

1. Twenty‐four meta‐programs and forty‐two non‐epithelial cells subtypes were identified in gastrointestinal tract cancer using over one million cells.
2. Esophageal cancer (EC), gastric cancer (GC) and colorectal cancer (CRC) display unique epithelial characteristics influenced by specialized tumor microenvironment.
3. GC and EC with a metaplastic phenotype demonstrate overlapping features with CRC.

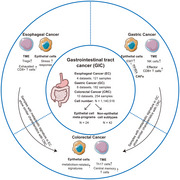

## 1 INTRODUCTION

Gastrointestinal tract cancer (GIC) exhibits a high incidence and mortality rates, accounting for approximately 17% of new cancer cases and 20% of cancer‐related deaths worldwide by 2022.^1^ The major types of GIC including oesophageal cancer (EC), gastric cancer (GC) and colorectal cancer (CRC), of which specific metaplastic transformation can be observed and considered as a pre‐cancerous stage. The heterogeneity and similarities of GIC have been extensively described at the genomic,^2^ transcriptomic,^3^, ^4^, ^5^ and epigenomic^6^ levels. The molecular subtypes spanning the three cancer types were identified including hypermutated‐single‐nucleotide variant predominant, microsatellite instability, chromosomal instability and genome stable, which displayed the shared features of GIC.^7^

The aforementioned researches have predominantly focused on features of malignant cells that were analysed at the tissue‐level, with limited concentration on the intratumour heterogeneity (ITH) of cancer cells and tumour microenvironment (TME) across different anatomical origins in GIC. ITH is closely associated with tumour progression and prognosis, particularly, because the diverse cellular subpopulations within a tumour contribute to its adaptability, promoting aggressive growth and resistance to treatment,^8^ ultimately affecting patient outcomes.^9^ Additionally, ITH affects non‐malignant compartments such as tumour‐infiltrating lymphocytes and cancer‐associated fibroblasts (CAFs) in the TME.^10^ scRNA‐seq studies have separately validated the existence of ITH and explored its diverse functions in EC,^11^ GC^12^ and CRC,^13^ potentially overlooking the inherent characteristics of GIC. Comparative analysis of ITH and their surrounding TME at the single‐cell level remains lacking.

ECs and GCs often originate in the context of metaplasia, but their formation occurs within distinct microenvironments.^14^, ^15^ Notwithstanding, ECs and GCs are managed with comparable therapeutic strategies, including PD‐1/PD‐L1 immune checkpoint inhibitors and CAR‐T cell therapy, both of which have demonstrated encouraging clinical efficacy across GIC.^16^, ^17^ Although the signature and similarity of metaplasia across gastrointestinal diseases have been well discussed,^18^ the resemblance of epithelial characteristics and immune microenvironments in GCs and ECs with metaplasia features and CRC have not been elucidated. Unravelling these connections could provide crucial insights into the shared mechanisms underlying the metaplasia‐associated characteristics.

By integrating and analysing public single‐cell RNA sequencing datasets, we examined the shared and distinguished meta‐programs (MPs) of epithelial cells in GIC and explored the relationship between these MPs and tumourigenesis. We investigated distinct epithelial cells within their surrounding microenvironments and identified potential interactions, revealing that the TME could critically reshape epithelial cell states. Similar characteristics of metaplastic samples from the GC and EC cohorts were observed with CRC samples. Together, this comparison offers new insights into the cellular and molecular heterogeneity of GIC, providing a foundation for targeted therapeutic strategies.

## 2 METHODS AND MATERIALS

### 2.1 Data collection

Public single‐cell transcriptomic datasets were obtained from several repositories, including the Gene Expression Omnibus (https://www.ncbi.nlm.nih.gov/geo/), EMBL‐EBI (https://www.ebi.ac.uk/biostudies/arrayexpress) and dbGAP (https://www.ncbi.nlm.nih.gov/gap/). The datasets included four EC datasets (GSE160269, GSE188900, GSE196756 and GSE199654), eight GC datasets (GSE134520, GSE150290, GSE163558, GSE167297, GSE183904, GSE184198, GSE206785 and phs001818.v1.p1) and ten CRC datasets (E‐MTAB‐8107, GSE132257, GSE132465, GSE144735, GSE161277, GSE166555, GSE178318, GSE178341, GSE188711 and GSE200997). All datasets were generated using 10× Genomics scRNA‐seq platforms, and primary samples without treatment were included in the analysis. Detailed information of these datasets was shown in Table S1.

Bulk‐RNA expression data, clinical data and somatic information from the cancer genome altas program (TCGA) were obtained from the portal (https://portal.gdc.cancer.gov/), which including four GIC cohorts (TCGA‐ESCA, TCGA‐STAD, TCGA‐COAD and TCGA‐READ). The v40 version was downloaded for the analysis.

### 2.2 Single‐cell RNA data processing

For public single‐cell RNA sequencing datasets, we first applied *Homo sapiens*. GRCh38.93 GTF file to standardise gene names. After formatting the gene names, we filtered out the cells identified as doublets using scDbiFinder (version 1.8.0)^19^ using a random mould. Additionally, cells with fewer than 500 detected genes or more than 20% unique molecular identifer (UMI) counts from mitochondrial genes were excluded due to low quality. Furthermore, cells with more than 6000 detected gene were excluded. Genes that were present in more than 10 cells were used for further analysis. The filtered matrices were processed to obtain normalised matrices using Scanpy (version 1.10.1).^20^

### 2.3 Unsupervised cell clustering, batch combating and cell type annotation

Each dataset was pre‐processed and clustered separately using the same pipeline. Highly variable genes (HVGs) were identified by calculating the normalised variance of each gene in the count data. For GSE206785, where count data were unavailable, we identified HVGs using a dispersion‐based method. Subsequently, the gene expression matrices were scaled after regressing out the effects of gene counts, UMI counts and the proportions of mitochondrial and ribosomal RNA. Principal component analysis (PCA) was performed using the HVGs. The top‐ranking principal components (PCs) were used for Leiden, a graph‐clustering method, and Uniform Manifold Approximation and Projection (UMAP) dimensional reduction was conducted for visualisation. Optimum parameters, such as the number of PCs and clustering resolution, were determined separately to optimise dimensionality reduction and clustering outcomes and showed in Table S2. All steps were performed using methods implemented in the Python package Scanpy.

An evident batch effect was observed when clustering GSE188900, GSE184198 and GSE206785 data. To correct the batch effect, we employed the Harmony^21^ algorithm with the default parameters and designed samples as the technical covariates. To evaluate the effectiveness of batch effect removal across all datasets, we calculated the silhouette scores using the function ‘silhouette_scores’ in sklearn.

To obtain differentially expressed genes (DEGs) for each cluster, we first used the ‘scanpy.tl.rank_gene_groups’ function in Scanpy to rank the genes. *p* values were calculated using the non‐parametric Wilcoxon rank‐sum test and adjusted *p* values were obtained based on the Benjamini–Hochberg procedure. DEGs were retained with the following criteria: an average expression at least 1.5 times higher than other groups, and an adjusted *p* value lower than .01. Clusters were annotated by combining DEGs with canonical cell type markers. Clusters that expressed canonical markers from two or more cell lineages were classified as doublet, while clusters without canonical marker expression were deemed as low‐quality clusters. Doublets and low‐quality clusters were both excluded from subsequent analyses.

### 2.4 Meta‐program construction

Following quality control, we excluded samples containing fewer than 50 epithelial cells, retaining 304 samples for MP construction. For each sample, non‐negative matrix factorisation (NMF; Snmf function in Nimfa [version 1.4.0]) was performed on a scaled expression matrix using the top 7000 genes ranked by gene expression. Mitochondrial and ribosomal genes were not included in the analysis, and the negative values in the scaled expression matrices were set to zero. The choice of K, which represents the number of components, can significantly influence the model's ability to uncover the heterogeneity of epithelial cells. We conducted NMF with *K* values ranging from 4 to 9. As a result, each sample generated 39 programs summarised by the top 50 genes with the highest NMF weight values. Furthermore, we define robust NMF programs based on three criteria:

Robust within the tumour: have several similar NMF programs within the same tumour. Two NMF programs were regarded as similar when they share at least 50% of signature genes.

Robust across tumours: show at least 20% similarity with any NMF programs from other tumours.

Non‐redundant within the tumour: the NMF programs were removed when they had more than 20% similarity with selected program in the same tumour.

After filtering out unique and redundant NMF programs, 1068 robust NMF programs were obtained. We performed Jaccard index‐based clustering of robust NMF programs to evaluate their similarity. Through manual curation, we established 24 MPs comprising 865 robust NMF components.

### 2.5 Meta‐programs functionally annotation

To determine the functions of the MPs, we conducted gene set enrichment analysis using the hypergeometric test in clusterProfiler::enricher() function (version 4.8.3).^22^ The annotated gene sets from msigdbr (version 7.5.1),^23^ specifically from the kyoto encyclopedia of genes and genomes (KEGG) canonical pathways (C2.CP.KEGG), Gene Ontology (C5.GO), cell type (C8) and hallmark (H) collections, were used for analysis. Signature genes in each MP were significantly enriched in annotated gene sets with an adjusted *p* value threshold lower than .05.

### 2.6 Meta‐programs abundance evaluation

The abundance of MPs in GIC was evaluated using statistical methods. For each MP in a specific cancer type, the observed number by dividing the number of NMF programs of the MP. The expected number was calculated using the number of robust NMF programs of the cancer types by the total number of robust NMF programs. The abundance was calculated using the following function and adjust *p* values were obtained through Bonferroni method.

$$A=\;\mathrm{lo}g_{2}\;\left(\frac{\mathrm{observed}\;+1}{\mathrm{expected}\;+1}\right)$$

We further classified abundance into four levels. The ‘absence’ of MP indicated that there was no MP‐related NMF program in a specific cancer type. Additionally, two thresholds, based on the values of *A*, were set at 1 and 2 to categorise the abundance levels as low, medium and high.

### 2.7 Calculating meta‐program activity and assigning epithelial cell identity

To decipher the characteristics of the identified MPs across samples, we applied the AUCell method (scPAFA.tl.fast_ucell_score function in scPAFA [version 1.11.3]), which is based on the area under the curve (AUC), to calculate the scores of MPs in epithelial cells.^24^, ^25^ The activity of a specific MP was represented by the median MP score in each sample, which was used for comparisons across different cancer types. Additionally, an alternative method using the scanpy.tl.score_genes was employed for validation.

To assign epithelial cells to a certain MP, the MP score matrix was normalised by mean subtraction for each MP. For each epithelial cell, the MP with the highest normalised score was designated as the identity of the epithelial cells.

To evaluate the preference exhibited by MPs within specific cancer types, we calculated the ratio of the observed to expected cell numbers (Ro/e) for each cancer type. The expected cell numbers for each MP were determined using the chi‐squared test. An MP was considered enriched in a specific cancer type if Ro/e > 1.

### 2.8 Construction of epithelial cell trajectories

To delineate the evolutionary relationships among epithelial cells in GICs, we constructed trajectories of different cancer types separately using Monocle2 (version 2.22.0). Three independent datasets (GSE199654, GSE134520, GSE161277) were selected for analysis after re‐annotation, as they collectively encompassed pre‐cancerous (e.g., metaplasia, dysplasia), normal and tumour samples.

Monocle2,^26^ an algorithm that employs reverse graph embedding, was used to order single cells in pseudotime. Genes expressed in more than 10% of the cells were selected, and the top 3000 HVGs were selected to construct a cell ordering trajectory. DDRTree, a dimensionality reduction method based on graph structure learning, was applied via the reduceDimension function with the selected genes, and trajectory construction was achieved using the orederCells function.

### 2.9 Data integration and non‐epithelial subtypes annotation

Non‐epithelial cells were extracted from each dataset for integration. GSE206785 which included only the normalisation matrix was excluded from further analysis. The top 3000 HVGs were identified for subsequent analysis. To correct the batch effects, we employed the scvi algorithm in scvi‐tools (version 1.1.2),^27^ which used a probabilistic model built by an expression matrix. The dataset was considered the source of the batch and the percentage of mitochondrial genes and ribosome genes were designed as continuous_covariate_keys to guarantee accurate harmonisation of data. To construct a neighbour graph and achieve visualisation, we applied ‘scanpy.pp.neighbours’ and ‘scanpy.tl.umap’ to the latent space constructed by scVI. Identification of DEGs, filtering low‐quality and doublet clusters and annotation clusters were performed as previously described.

### 2.10 Evaluation and comparison of cell subtype abundance

To figure out the alterations in the immune microenvironment during tumour progression and the unique TME among cancer types, we compared the absolute abundance and relative abundance of each cell subpopulation. The Ro/e was calculated to compare absolute abundance. The relative abundance was calculated by dividing it by the major cell type. Wilcoxon rank‐sum test and Benjamini–Hochberg test were performed to compare the relative abundance of cell subtypes between groups. Samples with fewer than 50 cells in certain major cell type were excluded to guarantee the reliability of the comparison results.

### 2.11 Construction of immune cells and epithelial cells hubs

To investigate the differences in immune microenvironments associated with epithelial cells that exhibit distinct MPs characteristics, we conducted unsupervised clustering analysis to discover the co‐existing patterns of immune cells and epithelial cells. A Pearson distance matrix was constructed based on the scaled relative abundances of cell subtypes using the classDiscovery:: distanceMatirx() function. This distance matrix was then used for hierarchical cluster on the basis of ‘ward.D’ method. Samples with fewer than 50 epithelial cells and immune cells were excluded from the analysis. Cell subtypes with fewer than 100 cells or more than 30% cells from a single sample were also filtered out.

### 2.12 Specific cell–cell communication of cell types in hubs

Liana+ (version1.3.0), a scalable framework that integrates multiple methods, was used to infer cell–cell communication between co‐existing cell subtypes.^28^ The analysis was conducted separately for samples with and without the hub, utilising the universal methods CellPhoneDB^29^ and CellChat,^30^ along with the CellPhoneDB database (v5.0.0). An aggregate of ligand–receptor scores was obtained using the ‘liana.method.rank_aggregate’ function. Epithelial cells were designed as ‘targets’ while immune cells served as ‘sources’. Ligands and targets expressed in more than 10% of the corresponding cells, and ligand–receptor pairs (*p* < .05) from both methods, were included for further comparison and visualisation. Network visualisation of cross‐talk between epithelial cells and the TME was generated via the R package igraph, with node layouts by the Kamada–Kawai algorithm.

### 2.13 Identification and annotation of CAFs

To identify CAFs from fibroblasts, we first performed hierarchical clustering based on the average gene expression in predefined clusters using the ‘scanpy.tl.dendrogram’ function. The correlation matrix was computed using the Pearson's method. CAF functions were annotated using gene set enrichment analysis, as previously described. The top 50 DEGs ranked by fold changes for each CAF cluster were utilised for analysis with the annotated gene sets from the KEGG canonical pathway (C2.CP.KEGG) and hallmark (H) collections in MsigDB.

### 2.14 Differentiation trajectory establishment of fibroblasts

We employed PAGA^31^ and Monocle2 to establish the differentiation trajectory of fibroblasts from normal fibroblasts (NFs) to CAFs Owing to the large size, random stratified sampling (50% of cells per cluster) was implemented prior to trajectory construction. PAGA, a graph‐based algorithm, was executed via ‘scanpy.tl.paga’ in Scanpy to infer cell populations connectivity, followed by recalculation of the nearest‐neighbour distance matrix and neighbourhood graph. The diffusion map space was denoised using ‘scanpy.tl.diffmap’, and edges with connectivity <.15 were pruned to optimise graph visualisation. For Monocle2 trajectory inference, the top 1000 HVGs were selected.

### 2.15 Prediction of potential ligands

We performed NicheNet (version 1.1.1)^32^ which used a prior ligand‐target regulatory model to identify potential ligands in specific fibroblasts that have the ability to regulate epithelial cells in designated status. Epithelial cells were designed as ‘receiver’ while fibroblasts served as ‘sender’. Potential ligands were predicted using the ‘predict_ligand_activities’ function. The genes represented in the MPs were used as the target gene list.

### 2.16 Cell lineage inference in tumour epithelial cells

An annotated single‐cell human cell landscape was obtained using the R package scHCL (version 0.1.1),^33^ serving as a reference to identify the source of epithelial cells in GIC. Signatures related to epithelial cells were included in the analysis. The expression matrix was normalised by dividing the UMI count of each gene per cell by the total UMI counts. For a better comparison between the two matrices, log normalisation was applied in both.

We assigned cell lineage identities based on Pearson correlation analysis. First, we calculated the Pearson correlation coefficient (PCC) between the expression profiles of individual cells and the signature genes of reference cell types, generating quantitative estimates of lineage similarity. (1) For cells showing correlation (PCC > .3), we conducted lineage assignment based on cancer type that cells were assigned to lineages originating from the same cancer type when available; (2) When the best‐matched lineage derived from other GICs, we assigned the corresponding lineage identity; (3) Cells demonstrating low correlation coefficients (PCC ≤ .3) and lacking GIC signatures in their top five signatures were categorised as ‘Non‐GI’.

### 2.17 Evaluation cell type/statue activity in TCGA bulk samples

To validate MPs and non‐epithelial cell characteristics in GIC and evaluate their associated with prognosis, we performed single‐sample gene set enrichment analysis (ssGSEA) using the R package gsva (version 1.48.3).^34^ This analysis calculated the enrichment scores for each combination of samples and gene sets. Transcripts per million expression matrices from four TCGA cohorts were merged prior to be calculated, and only tumour and normal samples were included in the analysis.

### 2.18 Survival analysis in TCGA bulk samples

To investigate the implication of MPs and CAFs on patient outcomes, we performed survival analysis in TCGA bulk‐RNA samples using survminer packages (version 0.4.9) in R. Kaplan–Meier survival curves were calculated using the survfit() function, with visualisations created using the ggsurvplot() function. To determine the optimal cutting points for dividing tumour samples into two subsets, we employed the survminer::surv_cutpoint() function to perform dichotomy of the scores of cell type, setting the ‘minprop’ parameters to .3.

### 2.19 Statistical analysis

Data analysis was conducted on R (version 4.3.1) and python (version 3.9.12). Wilcox rank‐sum test was applied to compare MPs activity, cell subtype proportion and cell lineage proportion among groups. DEGs were also calculated using the Wilcox rank‐sum test and corrected using the Benjamini–Hochberg method. The correlation between different gene programs was evaluated using the PCC and the *p* value was adjusted using false discovery rate (FDR). Survival analyses were conducted using the Kaplan–Meier method, and statistical significance was assessed using the log‐rank test. Differences with *p* values <.05 or FDR < .05 were considered as statistically significant. Statistical significance was represented as *p* < .05 (*), *p* < .01 (**) and *p* < .001 (***).

## 3 RESULTS

### 3.1 Construction of meta‐programs in treatment‐naïve gastrointestinal tract cancer

**FIGURE 1:**
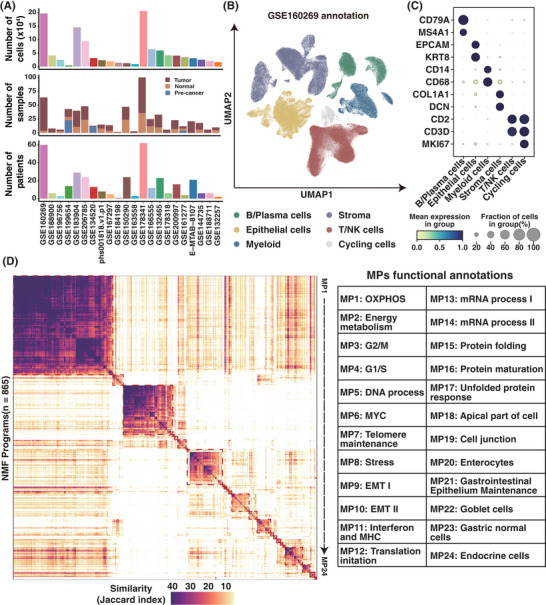
Meta‐programs (MPs) construction and annotation from scRNA‐seq datasets. (A) Summary of datasets collected in this study. The bar chart illustrates the number of cells, samples and patients in each dataset, displayed sequentially from top to bottom. (B) Unsupervised clustering and annotation of the GSE160269 dataset. Uniform Manifold Approximation and Projection (UMAP) visualisation of cells, coloured by cell types. (C) Validation of major cell types based on the proportions and scaled relative expression of canonical marker genes. Colours represent scaled normalised gene expression, while dot sizes reflect the fraction of each gene in the corresponding cell type. (D) Left: Heatmap depicting Jaccard similarity indices, computed using the top 50 genes from robust non‐negative matrix factorisation (NMF) programs. Programs are grouped into MPs, delineated by red‐dashed lines. Right: Functional annotation of all identified MPs.

To systematically compare the characteristics of ITH and immune microenvironment in GIC, we collected 22 public single‐cell RNA sequence datasets from previous studies including ECs, GCs and CRCs (Figure 1A). Only treatment‐naïve primary tissues, adjacent normal tissues and pre‐cancer lesions were involved. Altogether, 121 samples from EC, 182 samples from GC and 254 samples from CRC were obtained, of which 61% were primary tumour samples. We conducted clustering analyses for each dataset and implemented batch effect correction using the Harmony algorithm on three datasets (GSE188900, GSE184198, GSE206785; Figure S1). Quantitative assessment and dimensionality reduction visualisation collectively demonstrated that all datasets were free of significant batch effects (Figure S2). Following rigorous quality control and cell type annotation, we successfully obtained a total of 1 140 516 high‐quality cells, which were classified into six major cell lineages using canonical markers, including B and plasma cells, epithelial cells, myeloid cells, stromal cells, T and NK cells and cycling cells (Figures 1B,C and S3).

The epithelial cells of each sample were decomposed separately using a NMF‐based method to obtain modules defined by the genes with the highest weights. Then, unsupervised clustering and manual grouping were used in constructing consensus MPs through shared genes of the modules. After filtering out repetitive or sample specific modules, 865 modules were retained from 304 tumour samples and classified into 24 MPs (Figure 1D).

The MPs were annotated using gene sets derived from the most frequently shared genes within the modules in each MP (Table S3). Consistent with previous studies,^35^, ^36^, ^37^ our results confirmed the presence of common programs which represented for cellular metabolism such as respiration (MP1, MP2), cell cycle (MP3, MP4, MP5) and stress‐related (MP8, MP17). The programs (MP9, MP10) relevant to epithelial–mesenchymal transitions (EMTs) were identified, reflecting the dynamic phenotypic shifts in epithelial cells. Immune‐related program (MP11) was also observed, characterised by major histocompatibility complex (MHC) class II (CD74, HLA‐DRA). The programs referred to epithelial lineage of gastrointestinal tract were also detected, such as enterocytes (MP20), gastrointestinal epithelium maintenance (MP21), goblet cells (MP22), normal gastric epithelial cells (MP23) and endocrine cells (MP24; Figure S4). In summary, MPs with distinct functional profiles and cell lineage characteristics provide a comprehensive profile of ITH in GIC.

### 3.2 The distributions and frequencies of meta‐programs in gastrointestinal tract cancer

**FIGURE 2:**
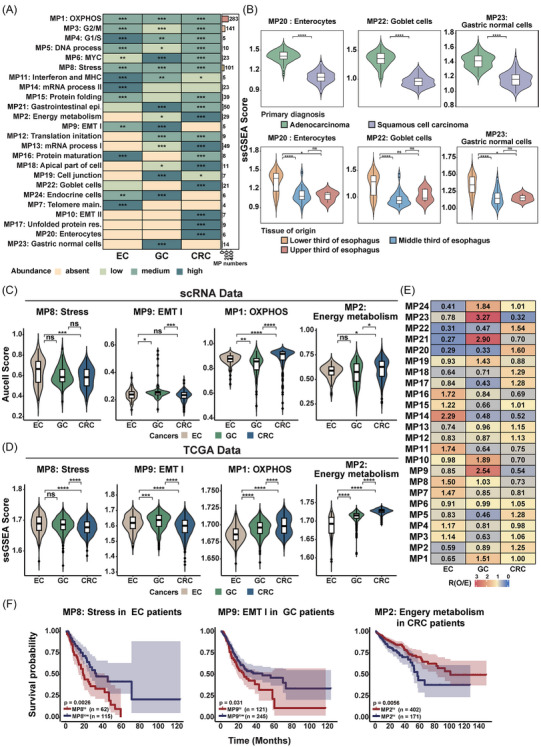
Meta‐programs (MPs) distribution and expression pattern in gastrointestinal tract cancer. (A) Left: Abundance of each MP across cancer types, categorised as absent, low, medium, high or significant, as defined by the described method. Right: Number of robust non‐negative matrix factorisation (NMF) programs associated with each MP. (B) Comparison of MP20 (enterocytes), MP22 (goblet cells) and MP23 (normal gastric cells) activities in oesophageal cancer samples from TCGA, grouped by clinical information. Top: Grouped by primary diagnosis (adenocarcinoma, *n* = 87; squamous cell carcinoma, *n* = 89). Bottom: Tissue of origin (upper third of oesophagus, *n* = 5; middle third of oesophagus, *n* = 43; lower third of oesophagus, *n* = 122). Boxplots show the median ± interquartile range, with whiskers representing 1.5× the interquartile range, and the centre line indicating the median. (C) Comparison of MP activity across cancer types in scRNA‐seq data. Violin plots showing the distribution of the median AUCell scores of epithelial cells within samples, grouped by cancer type. From left to right: MP8 (Stress), MP9 (EMT I), MP1 (OXPHOS) and MP2 (Energy metabolism). The number of samples: oesophageal cancer (EC; *n* = 75), gastric cancer (GC; *n* = 63), colorectal cancer (CRC; *n* = 155). (D) Comparison of MP activity across gastrointestinal tract cancer (GIC) cancer types in TCGA patients. Violin plots showing the distribution of ssGSEA calculated activities for each sample, grouped by cancer type. From left to right: MP8 (Stress), MP9 (EMT I), MP1 (OXPHOS) and MP2 (Energy metabolism). The number of samples: EC (*n* = 184), GC (*n* = 403), CRC (*n* = 619). (E) Prevalence of epithelial cell subpopulations across cancer types. The colour of the heatmap representing the value of Ro/e of each subpopulation in each cancer type. (F) Kaplan–Meier overall survival curves for patients stratified by MP activity. Left: MP8 (Stress) activity in EC patients; middle: MP9 (EMT I) activity in GC patients; right: MP2 (Energy metabolism) activity in CRC patients.

We first examined the distribution of MPs across datasets to evaluate the batch effect of dataset in MPs construction (Figure S5A). Except for MP7, the modules of all other MPs were derived from multiple datasets. Notably, only four samples exhibited the MP7 feature in that dataset, suggesting the MP7 was not caused by batch effects. Next, we explored the distribution and frequency of MPs among cancer types (Figures 2A and S5B). Oxidative phosphorylation (MP1), cell cycle (MP3) and stress‐related (MP8) were the most frequently identified in tumour samples. Two MPs (MP9 and 10) were annotated as EMT‐like patterns. Notably, MP10 was specific to CRC, while MP9 was detected in EC and GC, indicating the cancer‐type specificity of EMT process, which has been described in previous pan‐cancer research.^38^

MPs annotated as epithelial cell lineages were primarily linked to specific organs. The normal gastric cell lineage‐related signature (MP23) which is enriched with marker genes of chief cells and neck cells was specifically distributed in gastric tumour samples. Both enterocytes and goblet cells are intestinal epithelial cell types. The enterocyte signature (MP20) was restricted to CRC, whereas the goblet cell signature (MP22) was observed in both CRC and EC. Given the significance of metaplasia EC formation, TCGA data were used to examine the association of MP20, MP22 and MP23 with clinical features (Figure 2B). Oesophagus adenocarcinoma (EAC) exhibited higher levels of the programs compared to oesophagus squamous cell carcinoma (ESCC) consistent with it developed from Barrett's oesophagus. The lower oesophagus, which is more susceptible to intestinal metaplasia, also displayed higher scores than samples from other anatomical locations. Moreover, MP22 had opposite survival implications between CRC and EC cohorts in bulk TCGA samples (Figure S6A). Notably, although intestinal metaplasia is a pre‐cancerous stage in the development of intestinal‐type GC, we did not observe a significant difference in MP22 activity between intestinal‐type and diffuse‐type GC (Figure S6B).

### 3.3 Deciphering cancer‐specific upregulated expression pattern in gastrointestinal tract cancer

Further, we aimed to figure out whether MPs displayed different activity among different cancer types. Using AUCell to calculate (MP) activity scores for individual epithelial cells, we observed that certain MPs exhibited higher activity in specific cancer types (Figure 2C). For example, stress‐related signature (MP8) showed higher activity in EC, EMT‐like signature (MP9) was more active in GC, and metabolism‐related MPs, including oxidative phosphorylation signature (MP1) and energy metabolism signature (MP2), were elevated in CRC. The stress‐related MP was characterised by the expression of genes belonging to activator protein‐1 (AP‐1) family (e.g., FOS, JUN, JUNB) and heat shock proteins (HSPs) family (e.g., HSP90AA1, HSPA1B, HSPA1A). Higher expression levels of these genes were observed in EC. Meanwhile, increased expression of collagen molecules was detected in GC (e.g., COL5A1, COL6A1) which regarded as the signature of EMT‐like MP (Figure S7A). Additionally, we utilised various methods and datasets to validate. Initially, we used gene sets from MPs to evaluate the activity in tumours from the TCGA cohort, uncovering activity patterns in GIC that corresponded with trends observed in single‐cell RNA data (Figure 2D). Subsequently, we designated an MP identity for each epithelial cell to characterise its state. Ro/e analysis revealed that MP8 cells were predominantly enriched in EC, MP9 cells showed greater enrichment in GC, and MP2 cells were more prevalent in CRC (Figure 2E). Moreover, employing an alternative scoring method in single‐cell data, we obtained similar outcomes, further validating the reliability of our findings (Figure S7B).

Given the well‐established tumour heterogeneity across different histological subtypes, we examined whether our observed expression patterns were influenced by histological classification. Analysis of EAC samples in TCGA cohort revealed consistent MP patterns, indicating our key findings were not attributable to histological differences between squamous cell carcinoma and adenocarcinoma (Figure S7C). Meanwhile, the cancer‐specific upregulated expression pattern remained robust in TCGA cohort after exclusion of Epstein‐Barr virus （EBV) positive samples, suggesting minimal viral confounding effects on the overall results (Figure S7D). Furthermore, we assessed the prognostic significance of MPs in predicting patient survival outcomes, with a particular focus on MPs that display different activity in GIC. Using Kaplan–Meier analysis, we determined that MPs with elevated activity in specific types of GIC could act as prognostic indicators for these cancers. Specifically, EC patients with higher activity of the stress‐related MP (MP8) had poorer overall survival, and GC patients with increased activity of the EMT‐like MP (MP9) had worse survival outcomes (Figure 2F). CRC patients with higher activity of the energy metabolism‐related MP (MP2) showed longer overall survival, which was not observed in EC and GC patients (Figure S8A). Beyond stress and EMT characteristics, high expression of cell cycle signature has typically been considered as a poor prognostic factor for cancer.^39^ However, we found that the cell cycle signature could act as a positive survival indicator in GIC, particularly in GC and CRC samples (Figure S8B). This finding was further confirmed by the previously established cell cycle gene sets (Figure S8C).

### 3.4 Dynamic reprogramming of epithelial cell states in gastrointestinal carcinogenesis

**FIGURE 3:**
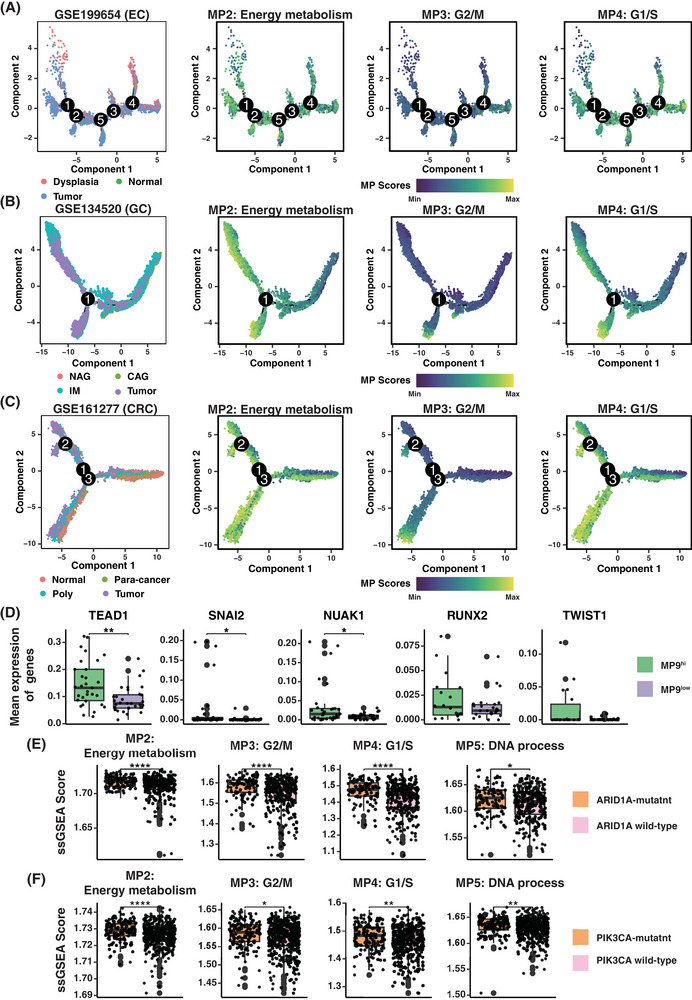
Genetic drivers and core regulatory modules orchestrate epithelial plasticity across gastrointestinal tumourigenesis. (A) Epithelial differentiation trajectory in oesophageal carcinogenesis from GSE199654 dataset. From left to right: coloured by sample types, MP2 (Energy metabolism) activity, MP3 (G2/M) activity, MP4 (G1/S) activity. (B) Epithelial differentiation trajectory in gastric carcinogenesis from GSE134520 dataset. From left to right: coloured by sample types, MP2 (Energy metabolism) activity, MP3 (G2/M) activity, MP4 (G1/S) activity. (C) Epithelial differentiation trajectory in colorectal carcinogenesis from GSE161277 dataset. From left to right: coloured by sample types, MP2 (Energy metabolism) activity, MP3 (G2/M) activity, MP4 (G1/S) activity. (D) Comparison of the expression level of epithelial–mesenchymal transition (EMT)‐associated transcription factors (TFs) between EMT‐high and EMT‐low groups from gastric tumour samples. From left to right: TEAD1, SNAI2, NUAK1, RUNX2 and TWIST1. The number of samples: EMT‐high (*n* = 31), EMT‐low (*n* = 32). (E) Comparison of activity scores of the metabolic signature (MP2) and cell cycle‐related meta‐programs (MPs; MP3, MP4, MP5) between ARID1A‐mutant and ARID1A wild‐type groups in gastric tumour samples from TCGA data. From left to right: MP2 (Energy metabolism), MP3 (G2/M), MP4 (G1/S), MP5 (DNA process). The number of samples: ARID1A‐mutant (*n* = 106), ARID1A wild type (*n* = 289). (F) Comparison of activity scores of the metabolic signature (MP2) and cell cycle‐related MPs (MP3, MP4, MP5) between PIK3CA‐mutant and PIK3CA wild‐type groups in colorectal tumour samples from TCGA data. From left to right: MP2 (Energy metabolism), MP3 (G2/M), MP4 (G1/S), MP5 (DNA process). The number of samples: PIK3CA‐mutant (*n* = 147), PIK3CA wild type (*n* = 426).

To delineate the evolutionary dynamics of MPs during gastrointestinal tumourigenesis, we constructed epithelial cell trajectories across three independent cohorts of EC (GSE199654), GC (GSE134520) and CRC (GSE161277; Figure S9A–C). Metabolism‐related (MP2), cell cycle‐related (MP3) and MYC signalling‐related (MP6) exhibited conserved activation patterns from pre‐malignant to invasive states across all three cancer types, underscoring their roles as core regulatory modules in carcinogenesis (Figure 3A–C). Meanwhile, we observed a downregulation of epithelial lineage‐related MPs (MP22, MP23) during malignant progression, indicating gradual loss of differentiation features during malignant transformation (Figure S9D). The coordinated upregulation of tumour‐promoting MPs and downregulation of lineage‐maintaining MPs across distinct gastrointestinal malignancies highlights the dynamic reprogramming of cellular states during carcinogenesis.

EMT is a pivotal mechanism promoting tumour invasion, metastasis and therapeutic resistance in cancer progression. TEAD1, SNAI2, NUAK1, RUNX2 and TWIST1 as key EMT‐associated transcription factors (TFs) were identified previously.^40^, ^41^ Considering of EMT‐like signature (MP9) exhibiting higher activity in GC, we stratified gastric tumours by EMT activity and observed elevated expression of those established TFs in the EMT high group (Figure 3D). The convergence of EMT signatures from single‐cell analysis and independent epigenomic datasets jointly demonstrates the pivotal role of this molecular network in GC progression.

Furthermore, we explored the potential effects of genetic mutations on epithelial cell status. ARID1A^42^ and PIK3CA,^43^ two driver genes with high mutation frequencies in GC and CRC, respectively, both demonstrated coordinated activation of proliferative and metabolic programs. In ARID1A‐mutant gastric samples and PIK3CA‐mutant colorectal samples, we observed concomitant elevation of cell cycle‐associated MPs (MP3, MP4, MP5) and energy metabolism signatures (MP2) (Figure 3E,F). These findings demonstrate that tumourigenesis‐associated mutations in ARID1A (GC) and PIK3CA (CRC) can influence epithelial cell state with proliferative and metabolic reprogramming, jointly contributing to gastrointestinal carcinogenesis.

### 3.5 Distinct tumour microenvironment features in gastrointestinal tract cancer

**FIGURE 4:**
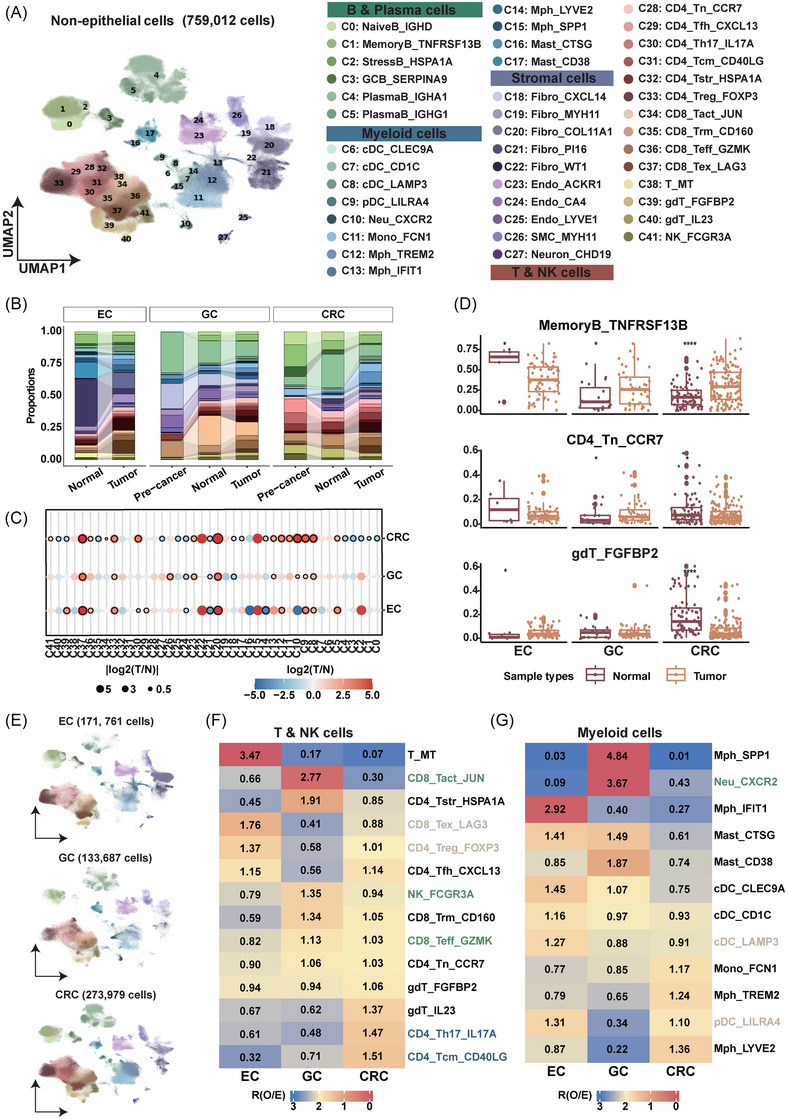
Microenvironmental landscape and characteristics in gastrointestinal tract cancer. (A) Unsupervised clustering and annotation of the non‐epithelial cells. Uniform Manifold Approximation and Projection (UMAP) visualisation of cells, coloured by cell types. (B) Alluvial plots illustrating the proportion of cell subtypes across sample types, grouped by cancer types. From left to right: oesophageal cancer (EC), gastric cancer (GC) and colorectal cancer (CRC). (C) Prevalence of each cell type across sample types, represented by a dot plot. The colour indicates the absolute value of the log₂ fold change in cell type proportions between tumour (T) and adjacent normal tissue (N) samples, while dot size reflects the log₂ fold change. (D) Box plot panels illustrating the tissue prevalence of specific cell subtypes in each cancer type. From top to bottom: memoryB_TNFRSF13B, CD4_Tn_CCR7 and gdT_FGFBP2. The number of samples from left to right: normal samples from EC (*n* = 7), tumour samples from EC (*n* = 70), normal samples from GC (*n* = 19), tumour samples from GC (*n* = 47), normal samples from CRC (*n* = 75), tumour samples from CRC (*n* = 155). (E) UMAP visualisation of non‐epithelial cells derived from tumour samples, grouped by cancer types. From top to bottom: EC, GC and CRC. (F) Heatmap showing the prevalence of T and NK clusters across cancer types. The colour of the heatmap represents the value of Ro/e of each cluster in each cancer type. (G) Heatmap showing the prevalence of Myeloid clusters across cancer types. The colour of the heatmap represents the value of Ro/e of each cluster in each cancer type.

The immune microenvironment comprises a dynamic network of immune cells, stromal cells and blood vessels which significantly influence cancer progression and therapy resistance.^44^ We first employed scvi‐tools for batch effect correction, which demonstrated superior capability to preserve the biological heterogeneity of cell populations with low abundance (Figure S11A). After re‐clustering, we obtained 49 non‐epithelial clusters after filtering low‐quality clusters and annotated 42 cell subtypes by DEGs and classic biomarkers (Figure S10 and Table S4).^45^, ^46^, ^47^, ^48^ There were four distinct B cell subtypes: naïve, memory, stress‐like and germinal centre B cells, as well as two plasma cell subtypes, IgA+ and IgG+ plasma cells. For T and NK cells, we identified naïve, exhausted, activated, effector, memory, stress‐like, helper and γδ T cells, along with one subtype of natural killer (NK) cells. In the myeloid lineage, we discovered four dendritic cell subtypes with varied functions, a neutrophil cell type, a monocyte cell type, four macrophage cell subtypes and two mast cell subtypes. Lastly, among stromal cells, we included several cell subtypes of fibroblasts, endothelial cells, smooth muscle cells and neuron cells (Figure 4A).

Firstly, we examined the distribution of cell subtypes across cancer types in GIC. Approximately half of the tumour‐infiltrating immune cells were specific subtypes of T cells and NK cells, underscoring their significant role in immune responses within the TME. IgA+ plasma cells (C4), which secrete IgA as a mucosal first‐line barrier,^49^ were primarily localised in adjacent normal tissues and pre‐cancer lesions of GC and CRC, but not in EC. Fibro_PI16 (C21) represented a substantial proportion in the adjacent normal tissues of EC, while Fibro_CXCL14 (C18) was predominantly found in GCs and CRCs, characterised by high expression of SOX6 and ADAMDEC1, suggesting these fibroblasts may be specific to the gastric and colonic microenvironments, as previously described^50^, ^51^ (Figure 4B).

Comparing with normal samples, the tumours exhibited an accumulation of exhausted T cells, including CD4_Treg_FOXP3 (C33) and CD8_Tex_LAG3 (C37). With respect to myeloid cells, cDC_LAMP3 (C8) and pDC_LILR4 (C9) were identified as being more prevalent in the TME. Dendritic cells expressing LAMP3 are likely mregDCs,^52^ which are implicated in the promotion of Tregs production,^53^ as well as plasmacytoid dendritic cells that may facilitate the recruitment of Tregs to the tumour site^54^ (Figures 4C and S11B,C). Notably, several cell subtypes displayed subtle inter‐cancer‐type differences. MemoryB_TNFRSF13B (C1) were more abundant in normal samples compared to tumour tissues of EC, a pattern that was reversed in normal and tumour samples from GC and CRC cohorts. Additionally, CD4_Tn_CCR7 (C28) and gdT_FGFBP2 (C39) exhibited a lower proportion in CRC tumour samples. Conversely, a higher proportion of CD4_Tn_CCR7 (C28) was observed in GC tumour samples, while gdT_FGFBP2 (C39) was more prevalent in EC tumour samples (Figure 4D).

We further explored the distinct characteristics of the TME across GIC (Figure 4E). T cells and NK cells, which displayed diverse states, reflecting the properties of the microenvironment (Figures 4F and S12A). Exhausted T cells such as CD8_Tex_LAG3 (C37) and CD4_Treg_FOXP3 (C33) were more abundant in EC samples, and no significant differences were observed between ESCC and EAC (Figure S12C). Previously research discovered that Tregs could inhibit the anti‐tumour immunity of γδ T cells which enrichment in EC tumour samples.^55^ Cell subtypes in an activated state, which directly kill tumour cells, like CD8_Tact_JUN (C34), NK_FCGR3A (C41) and CD8_Teff_GZMK (C36), were more prevalent in GC samples. While CRC samples have enrichment of CD4_Th17_IL17A (C30) and CD4_Tcm_CD40LG (C31). Furthermore, we confirmed the unique features of the TME across cancer types by demonstrating that immunosuppressive myeloid cell types, specifically cDC_LAMP3 and pDC_LILR4, were also enriched in EC (Figures 4G and S12B).

### 3.6 Diverse tumour cell characteristics and their specialised microenvironmental niches

**FIGURE 5:**
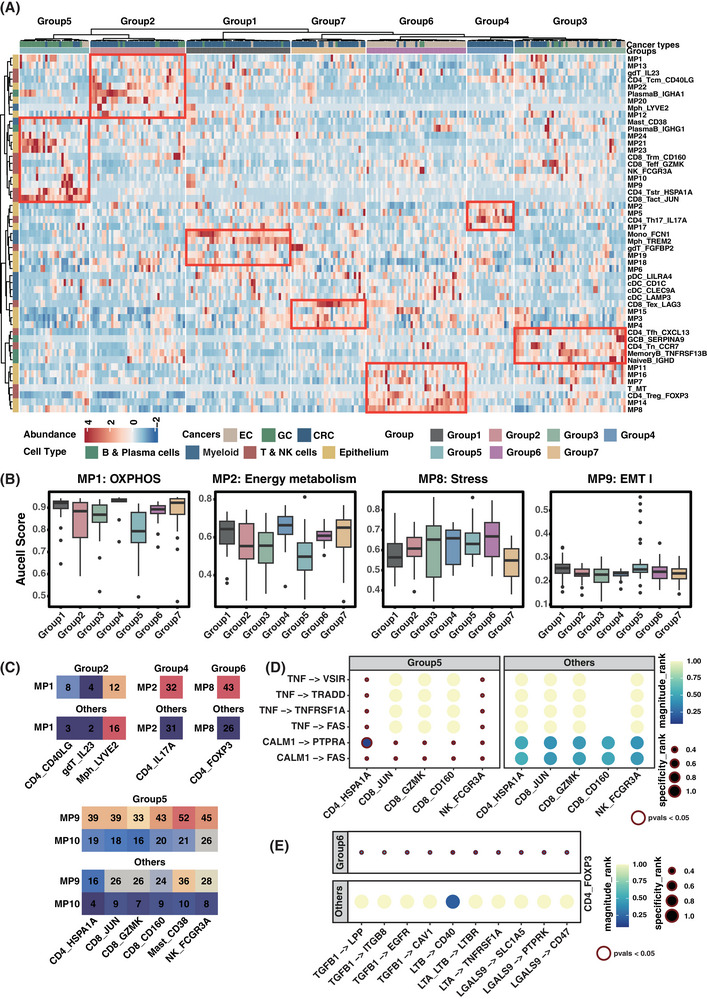
Microenvironmental niches and potential regulatory mechanism in gastrointestinal tract cancer. (A) Unsupervised clustering of samples based on the relative abundance of epithelial subpopulations and immune cell subtypes. The colour indicates the relative abundance of specific cell subtypes or subpopulations in the sample. (B) Boxplots showing meta‐program (MP) activity across different groups, calculated based on AUCell. From left to right: MP8 (Stress), MP9 (EMT I), MP1 (OXPHOS) and MP2 (Energy metabolism). The number of samples in each group: Group 1 (*n* = 45), Group 2 (*n* = 41), Group 3 (*n* = 48), Group 4 (*n* = 20), Group 5 (*n* = 30), Group 6 (*n* = 43), Group 7 (*n* = 32). (C) The number of ligand–receptor (LR) pairs between immune cell subtypes and epithelial cell subpopulations. The top of each panel displays the number of LR pairs in a specific group, while the bottom shows the LR pair counts in samples from other groups. (D) Estimation of reliable interactions from immune cell subtypes to epithelial cell subpopulations associated with MP9 (EMT I). Colour represents the expression level of interactions, while dot size indicates the specificity of interactions across all cell subtypes. (E) Estimation of reliable interactions from CD4_FOXOP3 cells to epithelial cell subpopulations associated with MP8 (Stress). Colour represents the expression level of interactions, while dot size indicates the specificity of interactions across all cell subtypes.

To investigate the immune microenvironment associated with specific tumour states and identify specialised microenvironment niches among cancer types, we applied the abundance of cell subtypes in the TME for clustering. Tumour samples were categorised as seven distinct groups, each characterised by a unique combination of immune cells and epithelial cells (Figure 5A). To account for batch effects originating from different datasets, we examined the distribution of samples from each dataset and observed that they were dispersed across distinct groups. Concurrently, each group contained samples from multiple datasets, with no single group being derived exclusively from one dataset. Notably, except for group 3, the remained groups were predominantly composed of samples from a specific cancer type that group 6 was primary composed of EC samples, group 5 mainly contained samples from GC and samples in group 1, group 2, group 4 and group 7 are predominantly from CRC. The distribution pattern of samples indicated the presence of distinct microenvironment niches among the different cancer types.

Further, we explored the relationship between cancer‐specific upregulated MPs in each group and their associated immune microenvironment. Stress‐like (MP8) epithelial cells were enriched in group 6, along with a high infiltration of CD4_Treg_FOXP3 (C33). Samples with a higher proportion of epithelial cells exhibiting EMT‐like MPs were primarily assigned to group 5, characterised by the co‐occurrence of CD8_Tact_JUN (C34) and stress‐like CD4_Tstr_HSPA1A (C32). Both oxidative phosphorylation (MP1) and energy metabolism (MP2) showed increased activity in CRC samples, while samples with a larger proportion of MP1 epithelial cells were categorised into group 2 and those with MP2 were assigned in group 4. When comparing MP activities between groups, MP1 had the highest score in group 4, but not in group 2 (Figure 5B). Additionally, CD4_Th17_IL17A (C30), which is enriched in CRC, was also observed in group 2 (Figure 4F), suggesting that group 2 is more likely to represent tumour‐specific niches of CRC compared to group 4. These observations indicate that epithelial cells with specific characteristics are surrounded by unique immune cell populations.

To depict cell–cell communication in specific microenvironment niches, we applied several methods to calculate cell–cell interactions between epithelial cells and immune cells. By analysing ligand–receptor interactions across all six groups (excluding group 3, which lacked MPs), we identified the interaction pairs between epithelial cells and TME that formed distinct interaction networks within each subgroup (Figure S13), within interaction profiles systematically showed in Table S5. Group 1 and group 5 exhibited the most wider interaction networks between epithelial and immune cells, with distinct cellular interaction patterns. Notably, myeloid cells (Mph_TREM2 and Mono_FCN1) formed the majority of ligand–receptor pairs in group 1, while T cell subsets (CD8_Trm_CD160, NK_FCGR3A) dominated the interaction profiles in group 5. This aligns with the notion that specific immune cell types may establish unique communication patterns within different TME niches, suggesting that the distinct cellular composition of each subgroup fosters particular interaction networks. Further comparative analysis revealed that stress‐like epithelial cells in group 6 samples exhibit greater interactions with exhausted CD4 T cells compared to the other groups. The higher level of cell interaction was observed in group 5 and group 2, indicating that the cell subpopulations in these niches are more active in communicating, potentially playing a crucial role in orchestrating immune responses (Figure 5C). Furthermore, we discovered that tumour necrosis factor (TNF) and calmodulin 1 (CALM1) secreted by immune cells were enriched in group 5 with their downstream genes, which may serve as a mechanism for inducing EMT of epithelial cells^56^, ^57^ (Figure 5D). Additionally, we observed that lymphotoxin alpha (LTA) and lymphotoxin beta (LTB) was significantly enriched in group 6. The ligand–receptor pair LTB‐CD40 were most connected in the group 6 (Figure 5E).

### 3.7 Fibroblast heterogeneity and impact on epithelial cells in gastrointestinal tract cancer

**FIGURE 6:**
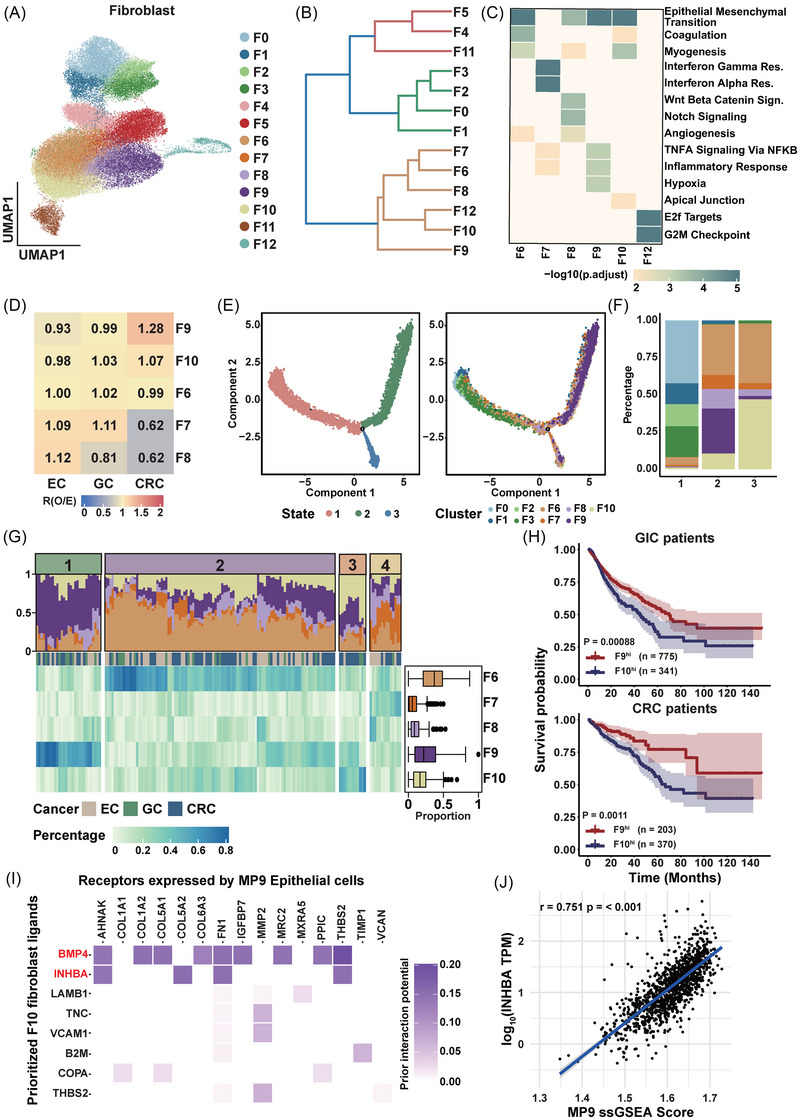
The heterogeneity, trajectory and regulatory role in cancer‐associated fibroblasts (CAFs). (A) Uniform Manifold Approximation and Projection (UMAP) visualisation of unsupervised clustering and annotation of fibroblast cells derived from tumour samples, coloured by clusters. (B) Unsupervised hierarchical clustering of 13 fibroblast clusters, coloured by classification categories. (C) Gene set enrichment analysis of CAF clusters. Heatmap displaying pathway enrichment for each cluster, with *p* values obtained via hypergeometric testing and adjusted using the Benjamini–Hochberg (BH) method, coloured by adjusted *p* values. (D) Heatmap showing the prevalence of CAF clusters across cancer types, with colour representing the Ro/e values for each cluster in each cancer type. (E) Inferred developmental trajectory of fibroblasts. Left: Trajectory coloured by cell states. Right: Trajectory coloured by fibroblast clusters. (F) Stacked bar plots showing the proportions of fibroblast clusters within each trajectory cell state. (G) Heatmap illustrating the proportions of CAF clusters in tumour samples. The top panel shows samples categorised into groups via hierarchical clustering, with CAF composition visualised in a stacked bar plot. Box plots on the right display the distribution of CAF proportions for each cluster. The samples involved in the analysis, oesophageal cancer (EC) sample (*n* = 61), gastric cancer (GC) sample (*n* = 26), colorectal cancer (CRC) sample (*n* = 71). (H) Kaplan–Meier overall survival curves for TCGA patients stratified by the gene signatures of F9 and F10. Top panel: Gastrointestinal tract cancer patients. Bottom panel: CRC patients. (I) Inferred top‐ranked potential ligands in F10 fibroblasts and their active target genes, coloured by predicted interaction potential. (J) Correlation between log10(INHBA TPM) and MP9 activity score across TCGA tumour samples. Each point represents an individual tumour sample (*n* = 1233). The solid blue line indicates the linear regression fit, with the shaded area representing the 95% confidence interval.

Fibroblast clusters were distributed with a preference across different cancers as previous described (Figure 4B). Thus, we tried to explore the features of fibroblasts in tumour samples of GIC and figure out their potential influence on epithelial cells. Fibroblasts from tumour samples were re‐clustered following Harmony‐based batch effect correction, yielding 13 clusters (Figure 6A). Comparative UMAP visualisation demonstrated superior dataset integration by Harmony over scvi‐tools, with minimal batch‐driven stratification (Figure S14A).

Firstly, we aimed to separate CAFs from clusters. Using hierarchical clustering we divided 13 fibroblast clusters into three categories: the first category included four clusters (F0, F1, F2 and F3), the second category comprised six clusters (F6, F7, F8, F9, F10 and F12) and the third category consisted of three clusters (F4, F5 and F11; Figure 6B). Using the marker genes of NFs and CAFs and DEGs of each cluster (Table S6), we identified the clusters in first category as NF‐like clusters with high expression of NFs makers such as DCN, MPG and IGFBP6. The CAFs makers including COL12A1, THY1, FAP, CAV1 and PDPN are elevated in second category^58^, ^59^ (Figure S14B). As for the third category, we found F4 and F5 were characterised by high expression of ADAMDEC1 and SOX6, respectively. F12 showed high expression of MYH11 and TAGLN but lacking RGS5 and PDGFRB was likely a vascular smooth muscle cell (vSMC) cluster^60^ (Figure S14D).

CAFs exhibit high heterogeneity and diverse functions, so we conducted a gene enrichment analysis using DEGs of CAF to depict their distinct features. We observed that F6 and F10 demonstrated a high level of myogenesis, however F10 showed elevated expression of smooth muscle markers like TAGLN and ACTA2, which were consistent with myofibroblasts, specifically designated as myofibroblast‐like CAFs (myCAFs). F7 was identified as interferon‐response CAFs (infCAFs) with the enrichment of interferon‐response pathways. The high expression of vascular‐related genes like HEY1 and NOTCH3 in F8 indicated the cell identity as vascular CAFs (vCAFs) with the enrichment in angiogenesis pathway. We designated F9 as inflammatory CAFs (iCAFs) with the DEGs of F9 enriched in inflammatory response and TNFα signalling like IL24 and IL6. F12 showed enrichment in mitotic spindle processes, indicating the cluster was dividing CAFs (dCAFs)^61^, ^62^ (Figures 6C and S14C). We observed iCAFs were more prevalent in CRC and vCAFs were enriched in EC (Figure 6D). The differences in CAF subpopulations further demonstrated the distinct TMEs in GIC.

To elucidate the evolutionary relationship among fibroblasts, we employed Monocle2 to construct a pseudotime trajectory with three distinct states. Our analysis revealed that NFs were primarily located in state 1, iCAFs are predominantly found in state 2 and myCAFs are more prevalent in state 3 (Figures 6E,F and S15A). Furthermore, when we clustered tumour samples based on the composition of CAFs, we found that samples with a higher proportion of iCAFs or myCAFs were classified into distinct groups, respectively (Figure 6G). Notably, there was an inverse relationship between the proportions of iCAFs and myCAFs in the samples (Figure S15B). These results suggested the existence of two distinct fibroblast differentiation trajectories in tumour samples, one leading towards iCAFs and the other tending towards myCAFs. By classifying cancer patients based on the enrichment of these two fibroblast categories, we found that the samples with a higher enrichment of iCAFs in GIC were associated with a better prognosis (Figures 6H and S15C), a finding consistent with previous observations.^63^

In the NF category, only F1 and F3 showed a connection with CAF subpopulations using PAGA (Figure S15D). Additionally, F1 was predominantly distributed in the transitional state 2 with the expression of the genes encoding pro‐inflammatory cytokine IL6 and chemokines CXCL2, further supporting its differentiation into iCAFs. These findings indicate that fibroblast remodelling occurs within NF‐like fibroblasts in tumours, with iCAFs and myCAFs deriving from distinct NF‐like fibroblast clusters.

To investigate the potential mechanism for CAFs regulating epithelial cells, we assessed the correlation between CAFs and MP activity in GIC. We observed a positive correlation between the proportion of myCAFs and EMT‐like MP9 activity (Figure S15E), both of which are associated with poor prognosis. Based on these findings, we infer that myCAFs may induce EMT, contributing to a worse clinical outcome. Using NicheNet, we identified potential ligands targeting EMT‐like MPs. Bone morphogenetic protein 4 (BMP4), a member of tumour growth factor beta (TGF‐β) family, and Inhibinβ‐A (INHBA) emerged as the top potential ligands (Figure 6I) that showed higher expression in myCAFs compared with iCAFs (Figure S16A). Furthermore, we found that INHBA exhibited a significant positive correlation with the EMT‐like MP9 activity in TCGA data (Figures 6J and S16B). Notable, INHBA expression also positively correlated with targets (e.g., AHNAK, COL5A2, FN1 and THBS2) and classic EMT markers (e.g., VIM, SNAI1 and TWIST1; Figure S16C). INHBA has been reported to induce EMT by activating TGF‐β regulated genes in breast cancer cell^64^ and promote of GC via targeting ITGA6 which contributes to EMT process.^65^ These findings suggested that INHBA may drive EMT, thereby contributing to a worse prognosis in GIC.

### 3.8 Gastric and oesophageal tumours within metaplastic lineages showing similar pattern with colorectal cancer

Metaplasia is a process triggered by environmental factors which is considered as a pre‐malignant stage in GC and EC. To investigate the impact of metaplasia on tumour characteristics, we first inferred potential cellular lineage of tumour cells in GIC using the well‐annotated human single‐cell atlas constructed previously as references.

**FIGURE 7:**
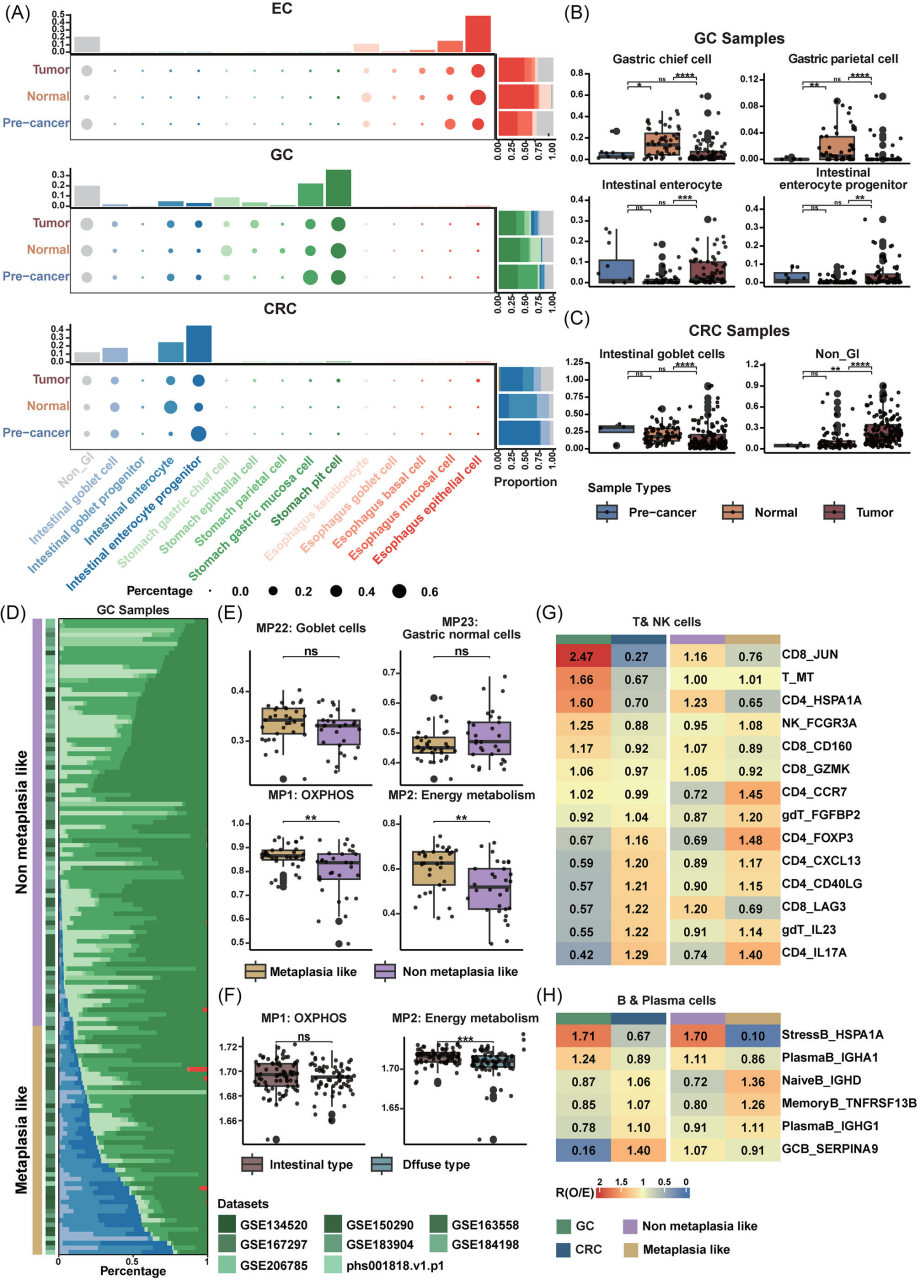
Similar pattern between metaplastic group in gastric cancer and oesophageal cancer with colorectal cancer. (A) Inferred cell lineage composition by comparing epithelial cell data to the HCL dataset. The dot plots show the composition of cell lineages (columns) across sample types (rows), with colour indicating identified cell lineages and dot size representing the fraction of each lineage within the sample type for a specific cancer type. The right panels display stacked bar plots of cell lineage composition by sample type, while the top panels show the proportions of specific cell lineages in each cancer type. From top to bottom: Oesophageal cancer, gastric cancer and colorectal cancer. (B) Comparison of cell lineage proportions among sample types. Top left: Gastric chief cell. Top right: Gastric parietal cell. Bottom left: Intestinal enterocyte. Bottom right: Intestinal enterocyte progenitor. The number of samples: pre‐cancer lesions (*n* = 12), normal samples (*n* = 53), tumour samples (*n* = 74). (C) Comparison of cell lineage proportions between normal and tumour samples in colorectal cancer. Top left: Intestinal enterocyte. Left: Intestinal goblet cell. Right: Non_GI (non‐gastrointestinal tract lineage). The number of samples: pre‐cancer lesions (*n* = 4), normal samples (*n* = 71), tumour samples (*n* = 155). (D) Stacked bar plots illustrating the proportions of cell lineages in each gastric cancer sample. Left annotations indicate the dataset and classification groups based on the proportion of intestinal cell lineages. The number of samples in each group: metaplastic group (*n* = 89), non‐metaplastic group (*n* = 50). (E) Comparison of MP activity between metaplastic and non‐metaplastic groups in tumour samples from gastric cancer. Top left: MP22 (Goblet cells). Top right: MP23 (Gastric normal cells). Bottom left MP1 (OXPHOS). Bottom right: MP2 (Energy metabolism). The number of samples in each group: non‐metaplastic group (*n* = 30), metaplastic group (*n* = 33). (F) Comparison of meta‐program (MP) activity between intestinal and diffuse types in tumour samples from gastric cancer samples in TCGA. Left: MP1 (OXPHOS). Right: MP2 (Energy metabolism). The number of samples in each group: intestinal‐type (*n* = 76), diffuse‐type (*n* = 66). (G) Heatmap showing the similar distribution pattern of T and NK subtypes between colorectal cancer and the metaplastic group in gastric cancer. Left: Prevalence of T and NK clusters between gastric cancer samples and colorectal cancer samples. Right: Prevalence of T and NK clusters between metaplastic and non‐metaplastic samples in gastric cancer. (H) Heatmap showing the similar distribution pattern of B and plasma subtypes between colorectal cancer and the metaplastic group in gastric cancer. Left: Prevalence of B and plasma clusters between gastric cancer samples and colorectal cancer samples. Right: Prevalence of B and plasma clusters between metaplastic and non‐metaplastic samples in gastric cancer.

In contrast to the majority of epithelial cells in CRC are classified as originating from intestinal tissue, the transcriptomic profiles of GC epithelial cells closely resembled cell feature not only from gastric but also from intestinal tissues, especially in tumour and pre‐malignant samples. This pattern aligned with the intestinal metaplasia is associated with the formation of GC. Meanwhile, a small population of cells in EC displayed goblet cell signature (Figure 7A). Through comparative analysis of lineage composition across sample types, we revealed an increase in enterocyte populations coupled with a reduction in gastric lineages (e.g., parietal and chief cells) within gastric tumour samples relative to normal tissues. Notably, pre‐cancer lesions exhibited lineage characteristics similar to tumours, attributable to intestinal metaplasia‐driven cell fate remodelling (Figure 7B). This suggests that enterocytes may act as pre‐malignant cells in the progression of GC. In CRC, goblet cells were more abundant in normal samples and pre‐cancer lesions, while the cells lacking gastrointestinal signature were more enriched in tumour samples (Figure 7C). This aligns with previous studies reporting goblet cell differentiation in tumourigenesis of CRC.^66^ In EC, the proportion of oesophageal epithelial cells and keratinocyte was decreased, while the population of mucosal squamous cells was increased in tumour samples compared with normal samples (Figure S17A).

Given the distinct composition of cell lineages, we categorised gastric samples into two groups based on the proportion of intestinal cell lineages (Figure 7D). The metaplasia group included not only tumour samples but also normal and pre‐cancerous samples. Notably, the proportion of intestinal cell lineages was higher in tumour samples compared to normal samples (Figure S17B), while the pre‐cancerous samples classified into the metaplasia group were those specifically at the intestinal metaplasia stage of pre‐cancerous progression. These observations suggest that the proportion of intestinal cells is progressively increased during tumour progression in GC. Compared to the non‐metaplasia group, the metaplasia group exhibited an elevated goblet cell signature (MP22) and a reduced gastric normal cell signature (MP23). Furthermore, in terms of MP activity, the metaplasia group demonstrated significantly increased activity in metabolism‐related MPs (MP1 and MP2) which also demonstrated heightened activity in CRC samples (Figure 7E). Using TCGA data, we observed that MP1 and MP2 were more active in intestinal‐type GC which developed from metaplasia compared to diffuse‐type GC (Figure 7F). Regarding immune cell composition, the metaplasia group exhibited the similar TME as in CRC samples with the enrichment of specific CD4+ T cell subtypes such as CD4_Th17_IL17A (C30) and CD4_Tcm_CD40LG (C31) and B cell subtypes like Naive B_IGHD (C0) and memory B_TNFRSF13B (C1) (Figure 7G,H).

Similarly, in EC samples, two groups were classified based on the proportion of metaplastic cells, which comprised gastric and intestinal cell lineages (Figure S17C). The metaplasia group in EC samples also displayed similar metabolism‐related MPs increase in activity and immune subtypes enrichment pattern (Figure S17D,E). CD4_Th17_IL17A was significantly enriched in metaplasia group, which is specific to CRC (Figure S17F,G). These findings indicate that both GC and EC samples harbouring metaplasia signatures share similarities with CRC in tumour characteristics and immune microenvironment.

In addition, we compared the features between metaplastic samples from EC and GC. Aligned with the exhausted microenvironment characteristic of EC, metaplasia samples from EC exhibited a more immunosuppressive profile than those from GC (Figure S18A,B). Meanwhile, EC metaplastic samples showed elevated MP8 activity, whereas the MP9 upregulation observed in GC samples did not differ significantly between EC and GC metaplastic samples (Figure S18C).

## 4 DISCUSSION

It is well established that tumour molecular characteristics, independent of their anatomical origin, guide immunotherapy and targeted therapy. For example, microsatellite instability‐high (MSI) a molecular subtype identified in GCs and CRCs, serves as a biomarker for immunotherapy. Similarly, the efficacy of immune checkpoint inhibitors depends on immune checkpoint molecule expression levels rather than tumour location.^67^ Therefore, comparative analyses across cancer types are crucial to unravelling tumour‐specific characteristics. A recent study by Chen et al. integrated gastric adenocarcinoma and adenocarcinoma of the oesophagogastric junction to provide insights into the TME in gastrointestinal cancers.^68^ However, such studies are often limited by small sample sizes and insufficient focus on ITH and the surrounding microenvironment.

In this study, we compared ITH and the TME at single‐cell resolution among EC, GC and CRC, identifying specialised microenvironmental niches and communication mechanisms. Within epithelial cells, we constructed 24 MPs reflecting diverse cellular processes, including tumour‐specific and ubiquitous activities. Among these, oxidative phosphorylation (MP1), cell cycle (MP3) and stress‐related programs (MP8) were the most prominent. The cell cycle, critical for rapid tumour cell proliferation, and the stress‐related program, induced by various stimuli and linked to drug resistance, were observed across multiple cancer types.^69^ The high occurrence rate of these MPs indicates that tumour cells require substantial energy for cell proliferation and are confronted with different stress such as hypoxia. Notably, we observed prevalent progressive activation of metabolism‐related (MP2), cell cycle‐related (MP3) and MYC signalling‐related (MP6) MPs during carcinogenesis, suggesting these conserved MPs may drive critical metabolic reprogramming, proliferative expansion and oncogenic transcriptional rewiring. These evolutionarily selected programs may collectively establish a molecular permissive niche for malignant transformation and therapeutic resistance.

We also identified MPs that are preferentially expressed in GIC. For example, EC exhibited increased activity in stress‐related programs (MP8), GC showed upregulation of EMT‐associated (MP9) and CRC demonstrated an elevated activity on metabolism‐related MPs (MP1 and MP2). These findings suggest that MPs may play distinct roles in different cancers, potentially driven by unique microenvironmental regulatory mechanisms. However, the immune microenvironment, a highly organised ecosystem, engages in bidirectional interactions with tumour cells. We identified 42 non‐epithelial cell subtypes and the distribution among cancer types is differed. EC harboured more in exhausted CD8+ T cells and Tregs, GC exhibited greater infiltration of cytotoxic effector cells such as NK and effector CD8+ T cells, and CRC showed higher levels of Th17 cells. Previous research also supported oesophageal tumour had more immune suppressive features compared with GC using flow cytometry.^70^ These observations emphasise how TME develop cancer‐specific features that influence epithelial states, providing insights for tailored therapeutic strategies.

We further constructed seven microenvironmental niches involving epithelial cells with specific states and their surrounding microenvironment, uncovering their communication mechanisms. Most niches comprised samples from a single cancer type, likely reflecting tumour‐specific microenvironmental conditions and cellular interactions. For instance, TNF, known to induce EMT via the NF‐κB pathway,^56^, ^71^, ^72^ and lymphotoxin, critical for Tregs proliferation and transendothelial migration, play roles in shaping epithelial and immune cell dynamics.^73^ Our study found that LTA potentially regulates stress‐like epithelial cells in the presence of Tregs, underscoring the interconnected regulation between immune cells and the epithelium.

CAFs further contribute to TME heterogeneity.^74^ We identified six distinct CAF subtypes, with iCAFs and myCAFs as predominant populations originating from different precursors. Notably, myCAFs secrete INHBA, which induce EMT and indicate poor prognosis.^75^ These findings position INHBA as a potential therapeutic target to disrupt myCAF‐driven EMT in gastrointestinal cancers. Neutralising INHBA (e.g., via monoclonal antibodies) or blocking its downstream ACVR1/SMAD2/3 signalling that have been investigated in vitro.^76^ Future studies should validate these approaches in pre‐clinical models stratified by CAF subtypes and explore combinatorial therapies with TGF‐β pathway inhibitors to amplify efficacy.^77^

Barrett's oesophagus and gastric intestinal metaplasia are regarded as molecularly similar pre‐cancerous lesions.^78^ Meanwhile, previous studies have demonstrated that tumour cells in GC originating from an intestinal lineage showed similar gene features with CRC.^79^ Building upon these findings, our results show that oesophageal and gastric tissues with metaplastic lineage closely resemble CRC, exhibiting higher metabolism‐related MP activity and Th17 cell enrichment. In inflammation models, intestinal epithelial cells promote naïve CD4+ T cell differentiation into Th17 cells, demonstrating how metaplastic cells reshape the immune microenvironment and adopt CRC‐like tumour characteristics.^80^ Although current evidence directly linking metaplastic GC/EC tumours to CRC‐specific therapies remains limited, their shared metabolic and immune features suggest potential therapeutic susceptibility. PSMB1, a signature gene in MP2, was identified as a therapeutic target in CRC, where the FDA‐approved drug Kinetin enhances PSMB1‐mediated oncoprotein degradation, effectively suppressing CRC progression in pre‐clinical models.^81^ Given that metaplastic GC/EC tumours exhibit elevated MP2 activity, where PSMB1 is a defining component, pharmacological activation of PSMB1 using Kinetin or analogous agents may represent a translatable strategy for these tumours. While further validation in GC/EC models is warranted, these findings highlight shared molecular vulnerabilities that could inform therapeutic repurposing strategies.

## 5 CONCLUSION

In this study, we integrated single‐cell data from gastrointestinal cancers to map epithelial MPs and non‐epithelial profiles. This approach elucidated tumour‐specific characteristics, regulatory mechanisms and similarities between CRC and metaplastic samples, paving the way for shared therapeutic strategies across GIC.

Despite the valuable insights gained, this study has several limitations. First, tumour heterogeneity, including variations in pathological subtypes, genetic backgrounds and clonal evolution, which may influence epithelial cells and TME characteristics. Although we accounted for potential confounders such as pathological subtypes in EC and EBV infection status using TCGA data, these adjustments may not fully capture the complexity of tumour ecosystems. Therefore, further validation in larger cohorts with single‐cell resolution and spatial transcriptomic profiling is essential to elucidate the differences in MPs, TME and niches in GIC. Meanwhile, the annotation of MPs is limited that the novel cellular states may miss due to reliance on prior knowledge. Second, we did not validate the influence of the immune microenvironment on epithelial cell features, such as CAFs secrete INHBA to induce EMT in epithelial cells. While computational analysis suggested potential interactions, experimental validation is essential to confirm the truly existence of these interactions and their modulating mechanisms. Finally, this study did not investigate whether EC and GC samples with metaplastic signatures might share similar treatment strategies with CRC. Further research is needed to explore the potential therapeutic overlap, which could provide new directions for treatment strategies across these cancer types.

## AUTHOR CONTRIBUTIONS

Chuwen Sun collected the data, performed the analysis, draft the manuscript and produced the figures. Tong Li offered valuable suggestions and revised the manuscript. Xin Jin, Zhihui Xiu and Hang Su provided feasible suggestions. Huanming Yang established the overall research goals and objectives. Ming Liu contributed to the design and conceptualisation of the study, supervised the research activities and provided funding support. Kui Wu conceived and designed the study, revised the manuscript, supervised the research activities and provided funding support. All the authors reviewed the manuscript.

## CONFLICT OF INTEREST STATEMENT

The authors declare no conflicts of interest.

## ETHICS STATEMENT

Not applicable.

## CONSENT FOR PUBLICATION

Not applicable.

## Supporting information



Supporting Information

Supporting Information

Supporting Information

Supporting Information

Supporting Information

Supporting Information

Supporting Information

## Data Availability

This study is based on publicly available data, and no new datasets were generated or analysed.
