# Supplementary material for: Integrative single‐cell transcriptomic analysis deciphers heterogeneous characteristics of gastrointestinal tract cancer
Source: Clin Transl Med. 2025 Aug 11;15(8):e70415. doi: 10.1002/ctm2.70415 (PMC12336674; doi:10.1002/ctm2.70415)
Supplement: Supplementary file 1 — Supporting Information [file CTM2-15-e70415-s006.docx]

**Supplementary Figures**

Supplementary Figure S1. Batch effect evaluation before and after Harmony integration across GSE188900, GSE184198, and GSE206785.

Supplementary Figure S2. Batch effect assessment for each dataset.

Supplementary Figure S3. Unsupervised clustering and annotation for each dataset.

Supplementary Figure S4. Annotation of each meta-program (MP).

Supplementary Figure S5. Evaluation of dataset-specific batch effects on meta-program detection.

Supplementary Figure S6. Association between MPs and clinical characteristics.

Supplementary Figure S7. Validation of MPs expression patterns in gastrointestinal tract cancer.

Supplementary Figure S8. Prognostic significance of meta-programs in gastrointestinal tract cancer.

Supplementary Figure S9. Epithelial differentiation trajectories in three independent datasets.

Supplementary Figure 10. Validation of non-epithelial subtypes via marker genes.

Supplementary Figure S11. Distribution of non-epithelial subtypes.

Supplementary Figure S12. Non-epithelial subtype comparison across cancers.

Supplementary Figure S13. Ligand-receptor networks in gastrointestinal tract cancer niches.

Supplementary Figure S14. Fibroblast clusters annotation.

Supplementary Figure S15. Fibroblast differentiation trajectories and prognostic subtypes associations.

Supplementary Figure S16. Association between INHBA and EMT signatures and marker genes.

Supplementary Figure S17. Conserved metaplastic patterns in gastric cancer and esophageal cancer with colorectal cancer.

Supplementary Figure S18. Comparative analysis of metaplastic features in esophageal and gastric cancers.


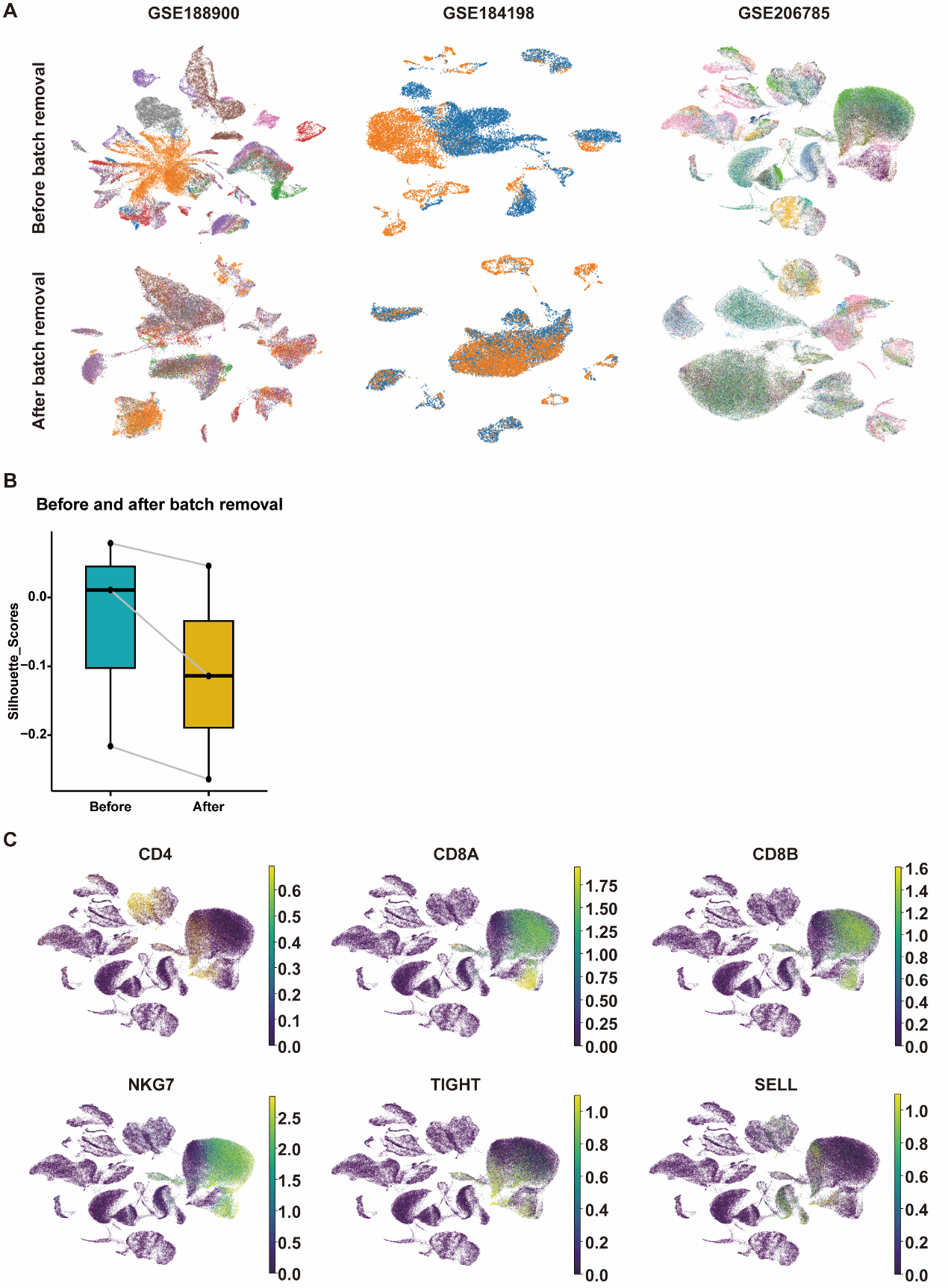


**Supplementary Figure S1. Related to Figure 1.** **(A).** Uniform Manifold Approximation and Projection (UMAP) visualization of GSE188900, GSE184198, and GSE206785 pre- and post-Harmony integration, colored by samples. **(B).** Comparison of silhouette score of GSE188900, GSE184198 and GSE206785 pre- and post-Harmony integration. **(C).** UMAP visualization of CD4, CD8A, CD8B, NKG7, TIGHT, and SELL in GSE206785, suggesting potential batch effect in this dataset.

**
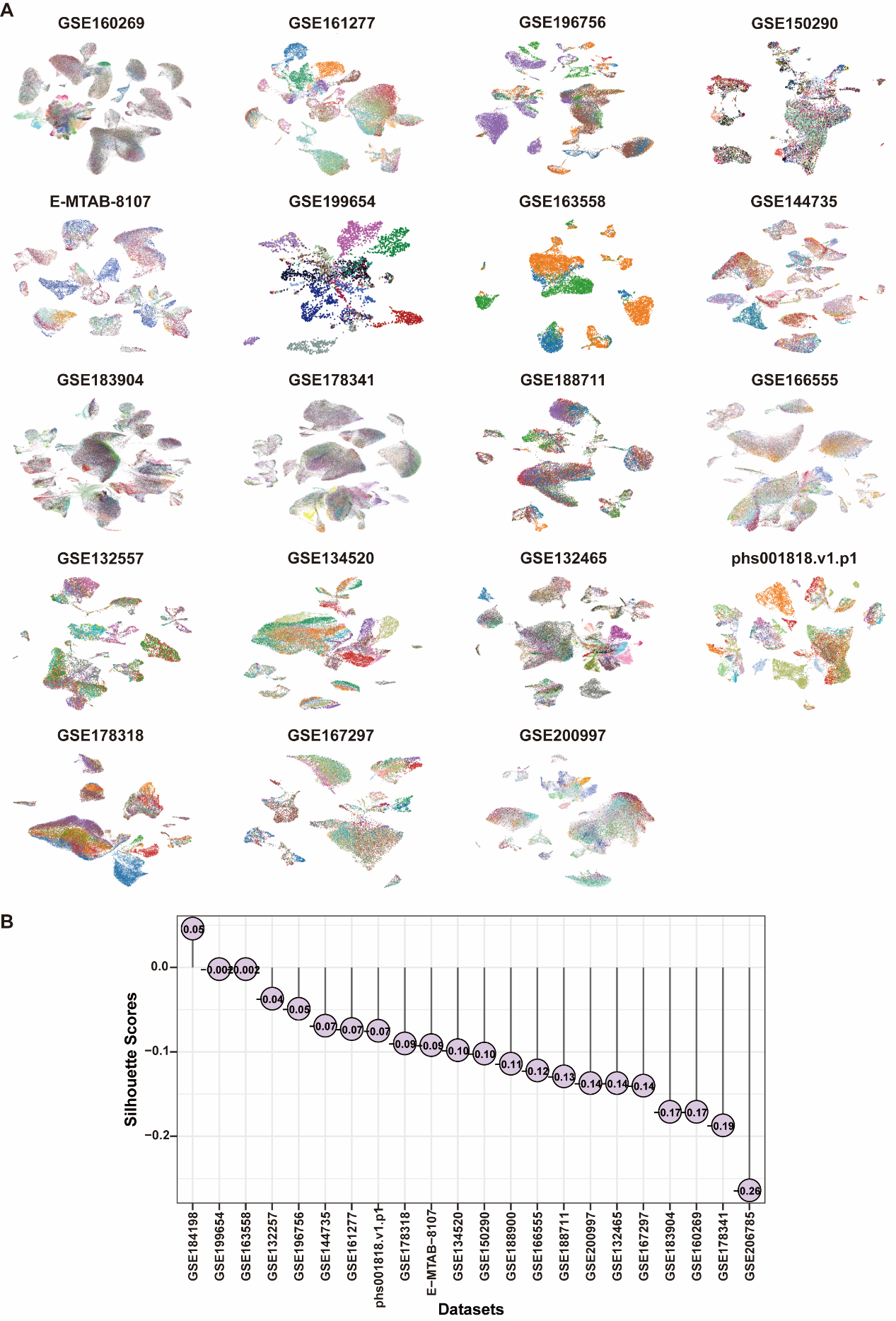
**

**Supplementary Figure S2. Related to Figure 1. (A).** Batch effect assessment by UMAP visualization, colored by samples. **(B).** Dot plots illustrating the silhouette score of each dataset.


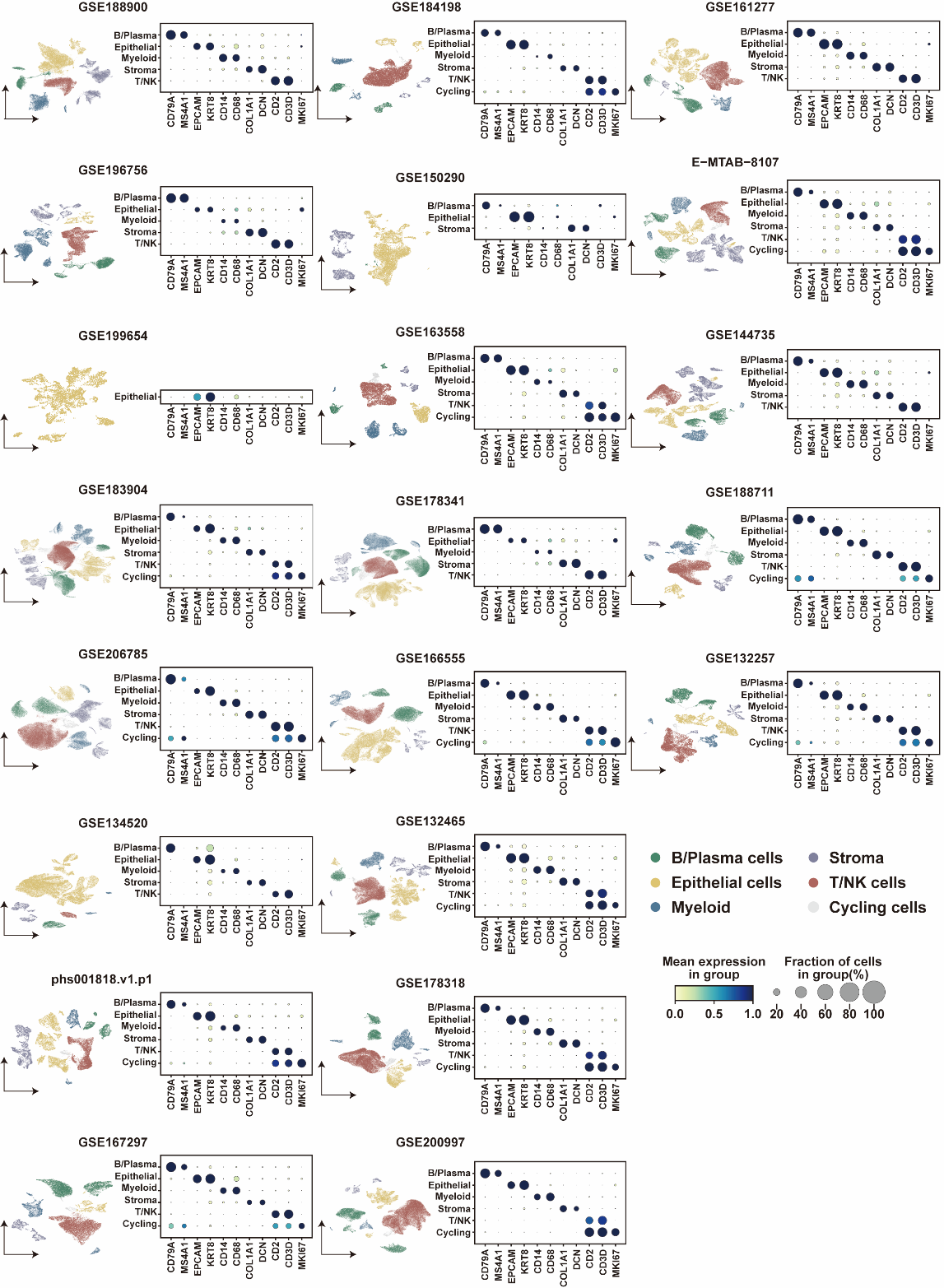


**Supplementary Figure S3. Related to Figure 1.** Left: unsupervised clustering and annotation of each dataset. UMAP visualization of cells, colored by major cell types. Right: validation of major cell types based on the proportion and scaled relative expression of canonical marker genes. Colors represent scaled normalized gene expression, while dot size reflects the fraction of each gene in specific cell type.


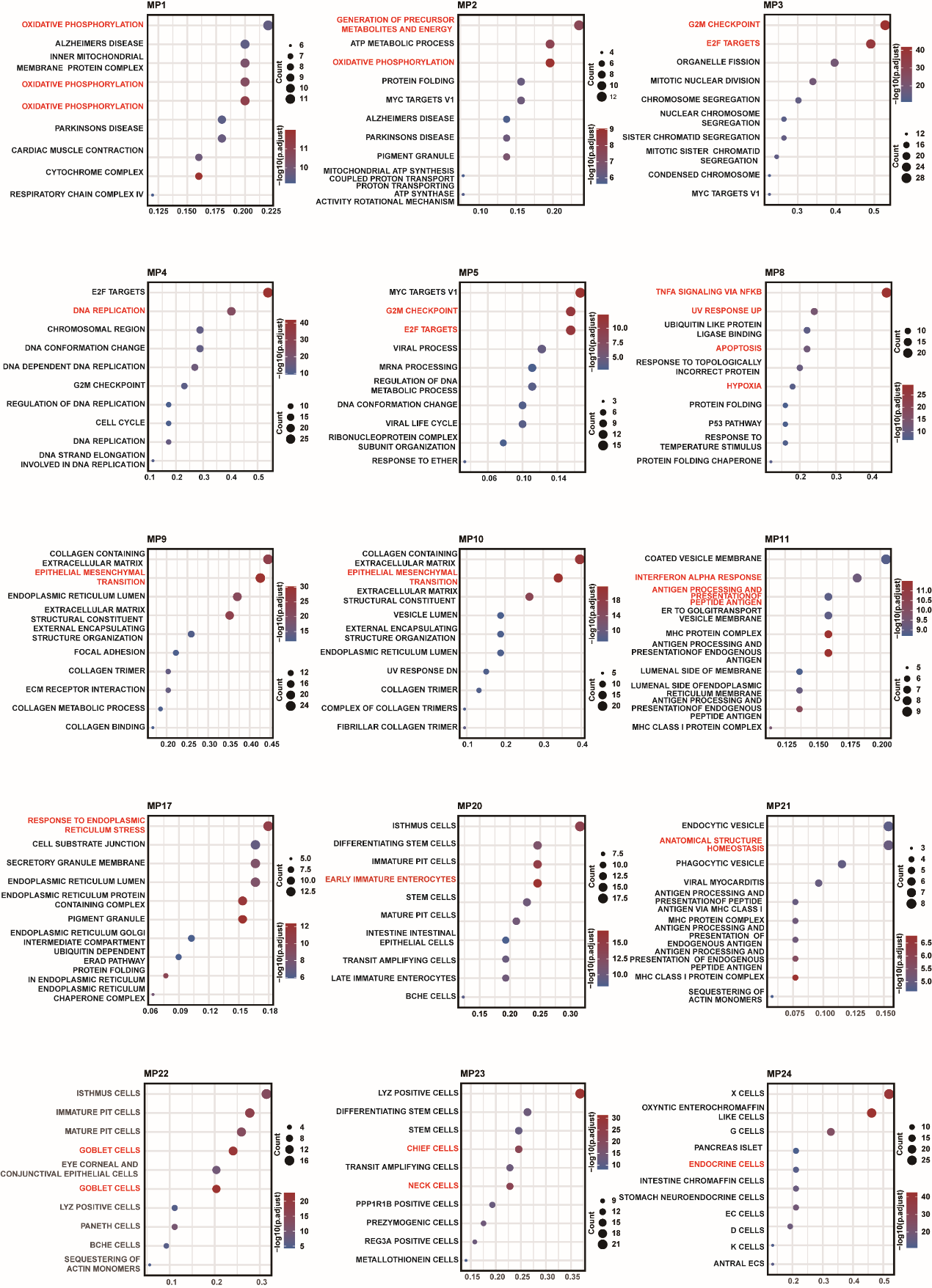


**Supplementary Figure S4. Related to Figure 1.** Dot plots illustrating the enrichment pathways for each specific MP, colored by adjusted p-values calculated using the Benjamini-Hochberg (BH) method after hypergeometric tests. Dot sizes represent the number of overlapping genes between meta-program signature genes and pathway genes. Functional pathways associated with the MPs are highlighted in red.


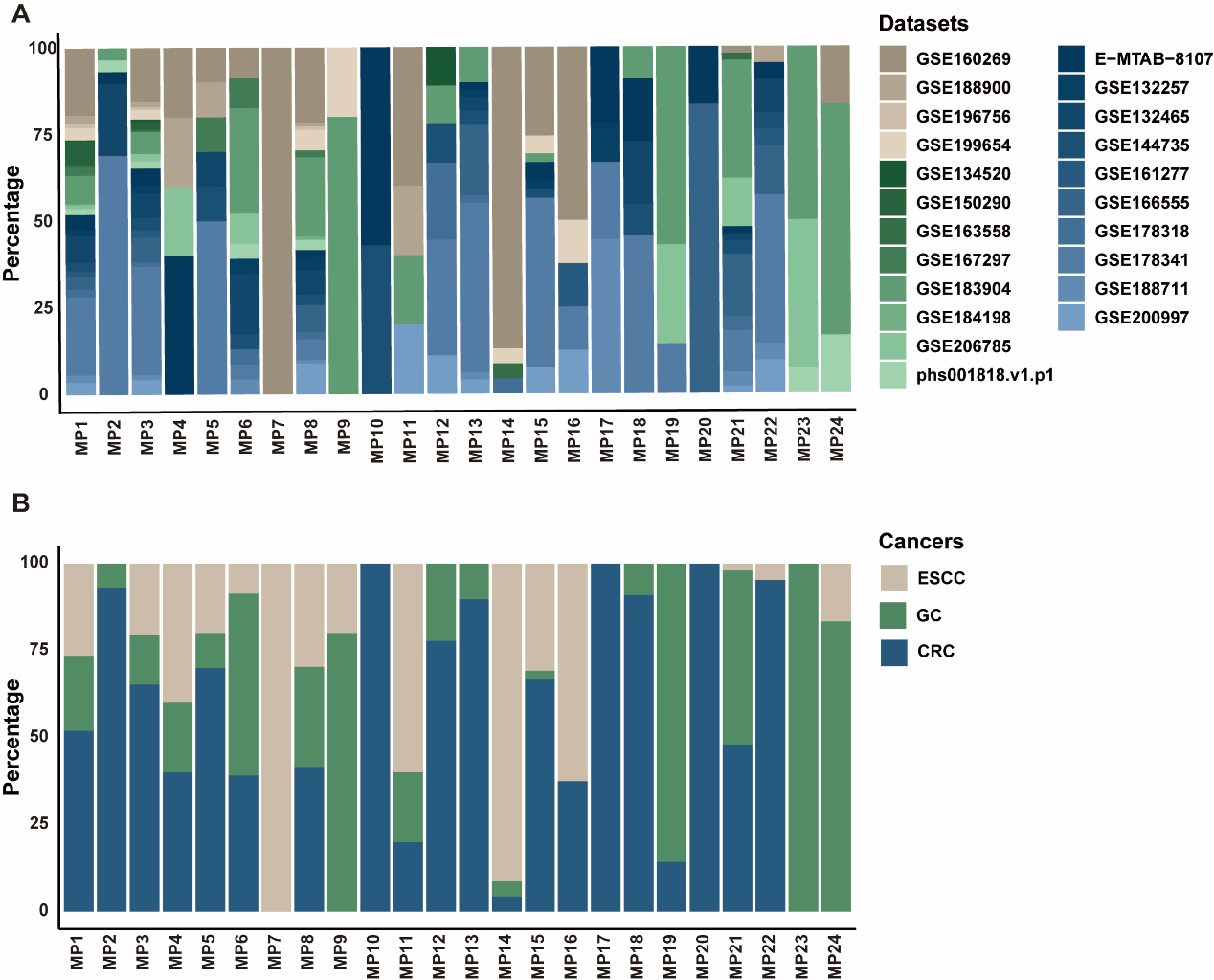


**Supplementary Figure S5. Related to Figure 2.** **(A).** Stacked bar plot showing the proportion of robust NMF programs contributed by each dataset within individual meta-programs (MPs), with colors corresponding to datasets. **(B).** Stacked bar plots displaying the proportion of robust NMF programs derived from different cancer types within each MP, where colors correspond to cancer types.


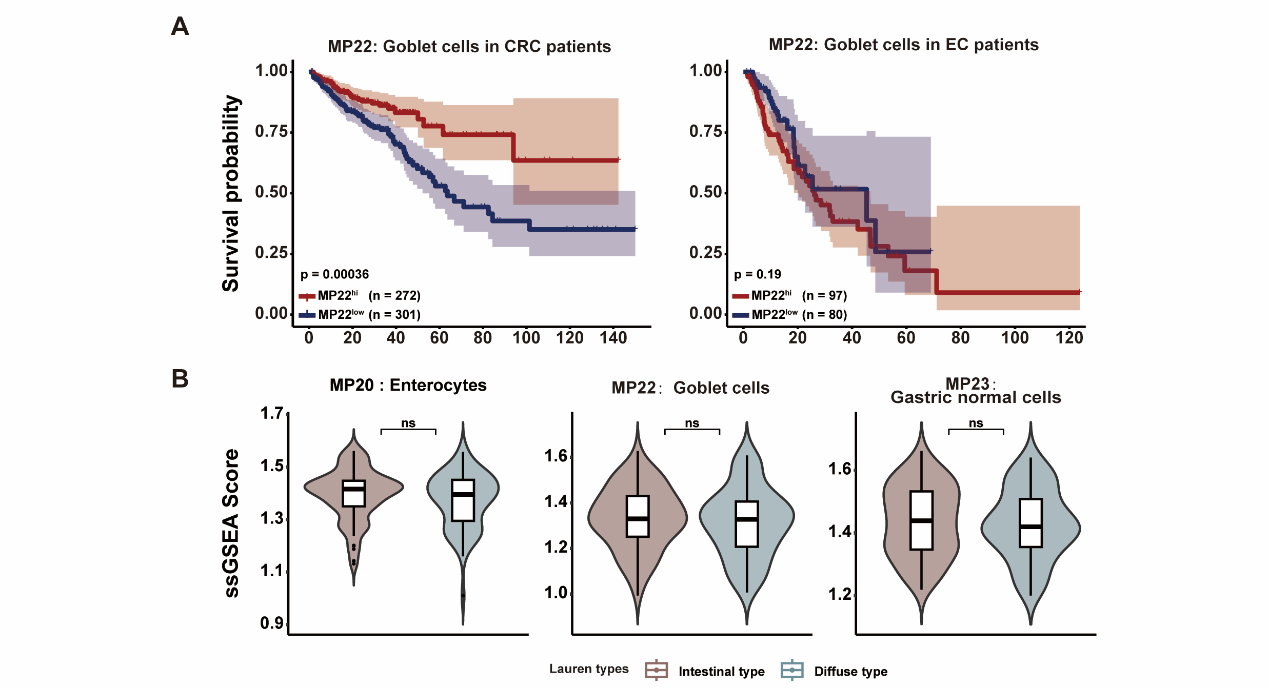


**Supplementary Figure S6. Related to Figure 2. (A).** Kaplan-Meier overall survival curves for TCGA patients stratified by MP22 (Goblet cells) signature. Left: colorectal cancer patients. Right: esophageal cancer patients. (**B).** Comparison of MP activity between intestinal and diffuse types in gastric cancer samples from TCGA dataset. Left: MP20 (Enterocytes). Middle: MP22 (Goblet cells). Right: MP23 (Gastric normal cells). The number of samples in each group: intestinal-type (n = 76), diffuse-type (n = 66).


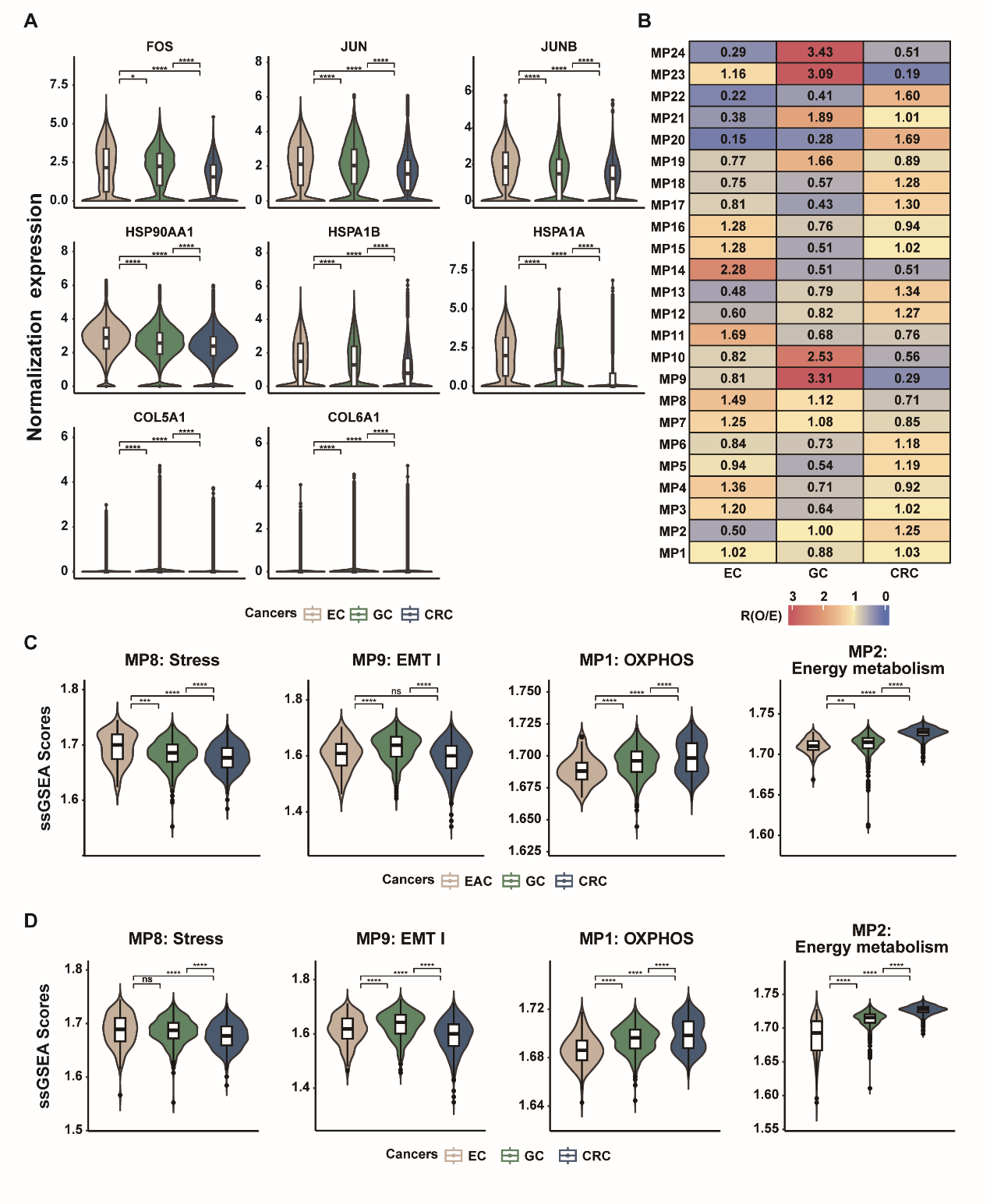


**Supplementary Figure S7. Related to Figure 2. (A).** Violin plots showing the expression of specific MP signature genes across cancer types. Top: FOS, FOSB, JUNB, associated with activator protein-1 (AP-1) family. Median: HSP90AA1, HSPA1B, HSPA1A, linked to heat shock proteins (HSPs) family. Bottom: COL5A1, COL6A1, related to collagen molecules. **(B).** Prevalence of epithelial subpopulation across cancer types. The heatmap colors represent the Ro/e value for each subpopulation in each cancer type. MP scores were calculated based on the average expression of their signature genes. **(C).** Comparison of MP activity across gastrointestinal adenocarcinomas cancer types in TCGA patients. Violin plots showing the distribution of ssGSEA activity for each sample, grouped by cancer types. From left to right: MP8 (Stress), MP9 (EMTI), MP1 (OXPHOS), and MP2 (Energy metabolism). The number of samples: EAC (n = 87), GC (n = 403), CRC (n = 619). **(D).** Comparison of MP activity across GIC in Epstein-Barr virus (EBV) negative TCGA patients. Violin plots showing ssGSEA activity distribution following removal of EBV positive samples, grouped by cancer types. The number of samples: EC (n = 184), GC (n = 342), CRC (n = 619).


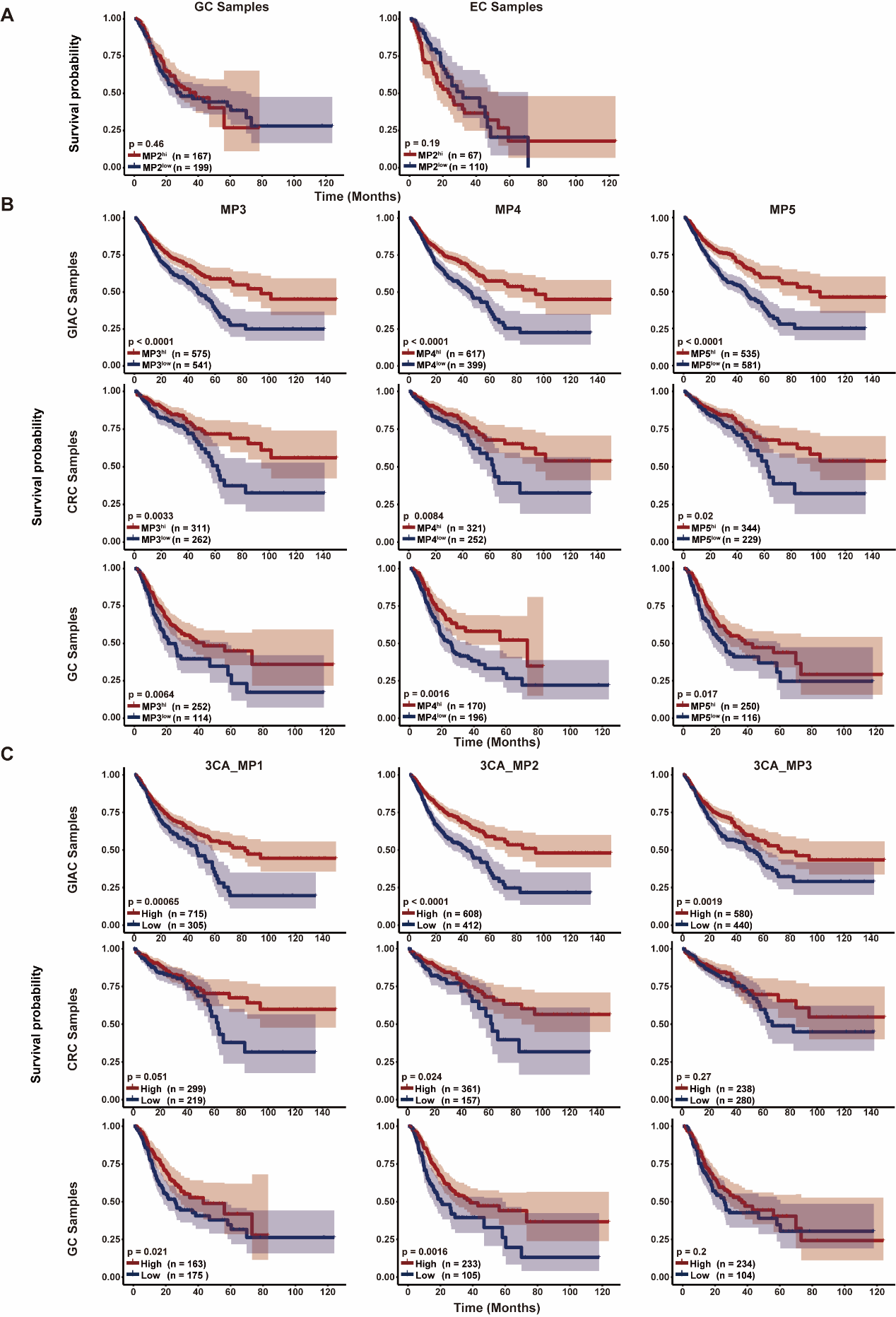


**Supplementary Figure S8. Related to Figure 2. (A).** Kaplan-Meier overall survival curves for TCGA patients stratified by MP2 (Energy metabolism). Left: gastric cancer patients. Right: esophageal cancer patients. **(B).** Kaplan-Meier overall survival curves for TCGA patients stratified by cell cycle related signatures. From left to right: G2/M (MP3), G1/S (MP4), and DNA process (MP5). From top to bottom: patients from gastrointestinal tract cancer, colorectal cancer, and gastric cancer. (**C).** Kaplan-Meier overall survival curves for TCGA patients stratified by cell cycle related signatures from 3CA. From left to right: G2/M (MP1), G1/S (MP2), and HMC rich (MP3). From top to bottom: patients from gastrointestinal tract cancer, colorectal cancer, and gastric cancer.

**
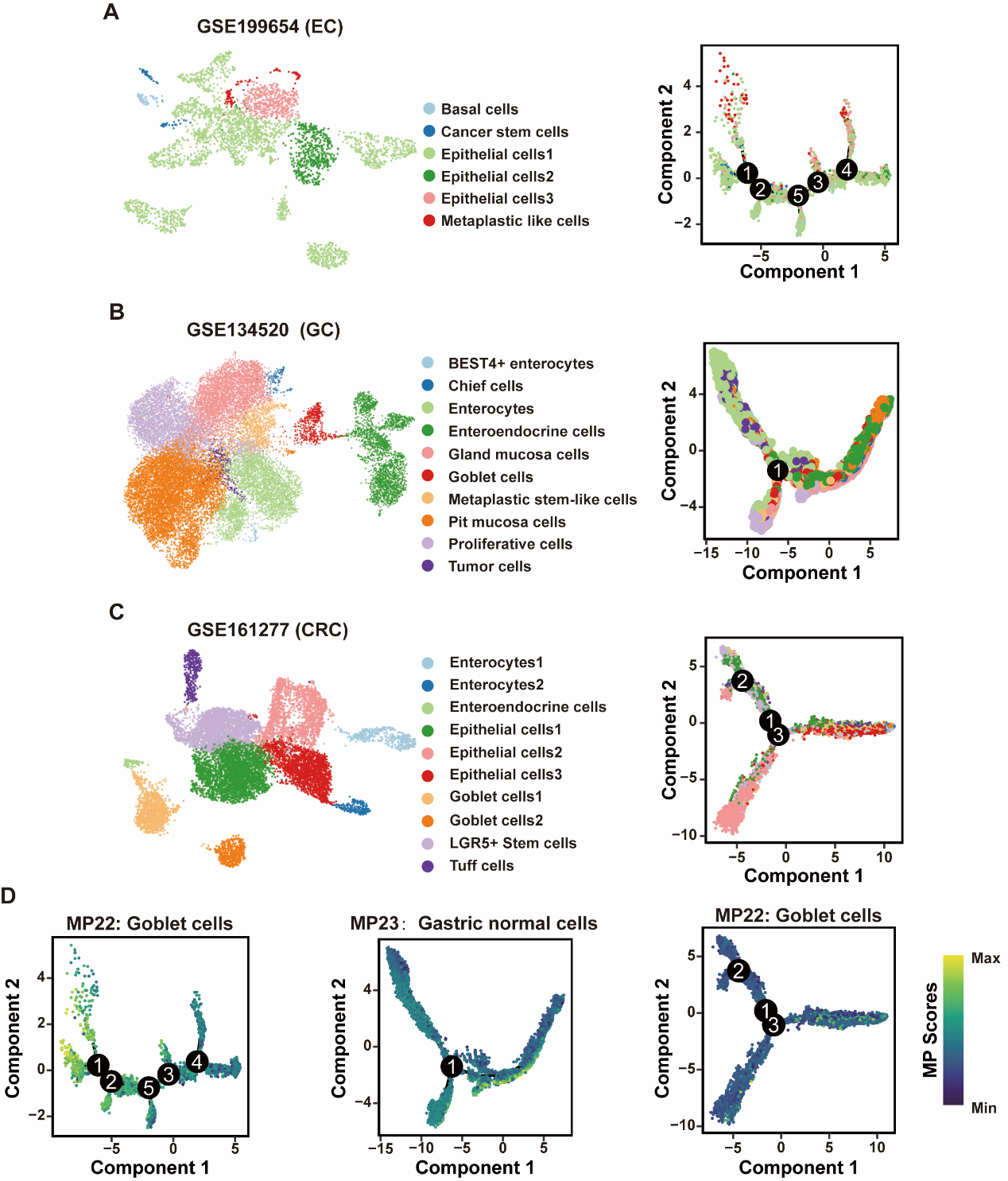
Supplementary Figure S9. Related to Figure 3. (A).** Left: UMAP visualization of annotation of epithelial cells from GSE199654 dataset, colored by cancer types. Right: Epithelial differentiation trajectory in esophageal carcinogenesis from GSE199654 dataset, colored by cell types. **(B).** Left: UMAP visualization of annotation of epithelial cells from GSE134520 dataset, colored by cancer types. Right: Epithelial differentiation trajectory in esophageal carcinogenesis from GSE134520 dataset, colored by cell types. **(C).** Left: UMAP visualization of annotation of epithelial cells from GSE161277 dataset, colored by cancer types. Right: Epithelial differentiation trajectory in esophageal carcinogenesis from GSE161277 dataset, colored by cell types. (D) Cell lineage related meta programs scores in epithelial differentiation trajectory. Left: MP22 (Goblet cells) activity in GSE199654. Middle: MP23 (Gastric normal cells) activity in GSE134520. Right: MP22 (Goblet cells) activity in GSE161277


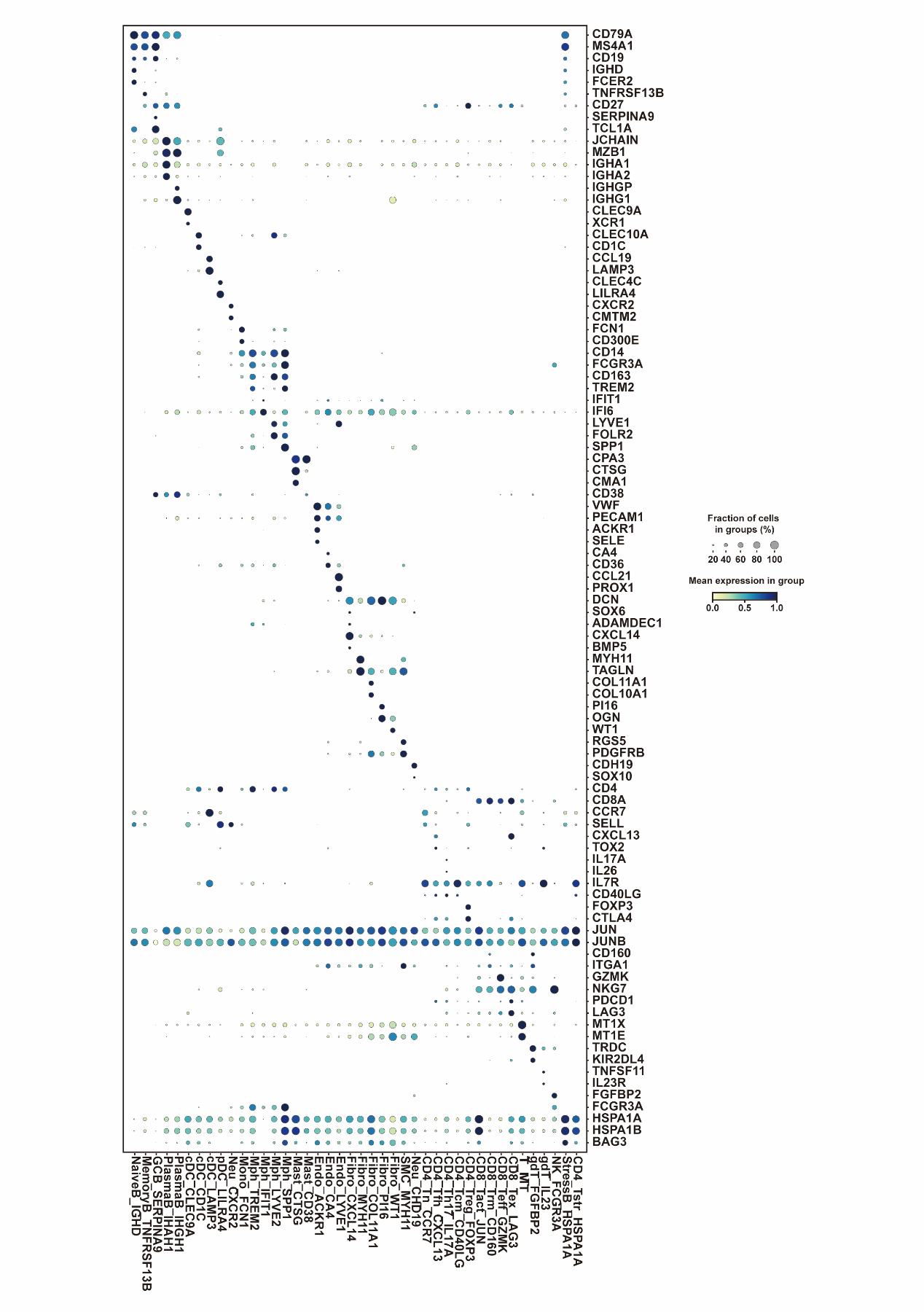


**Supplementary Figure S10. Related to Figure 4.** Validation of non-epithelial subtypes based on the proportion and scaled relative expression of canonical marker genes. Colors represent scaled relative gene expression, while dot sizes reflect the fraction of each gene in specific cell type.


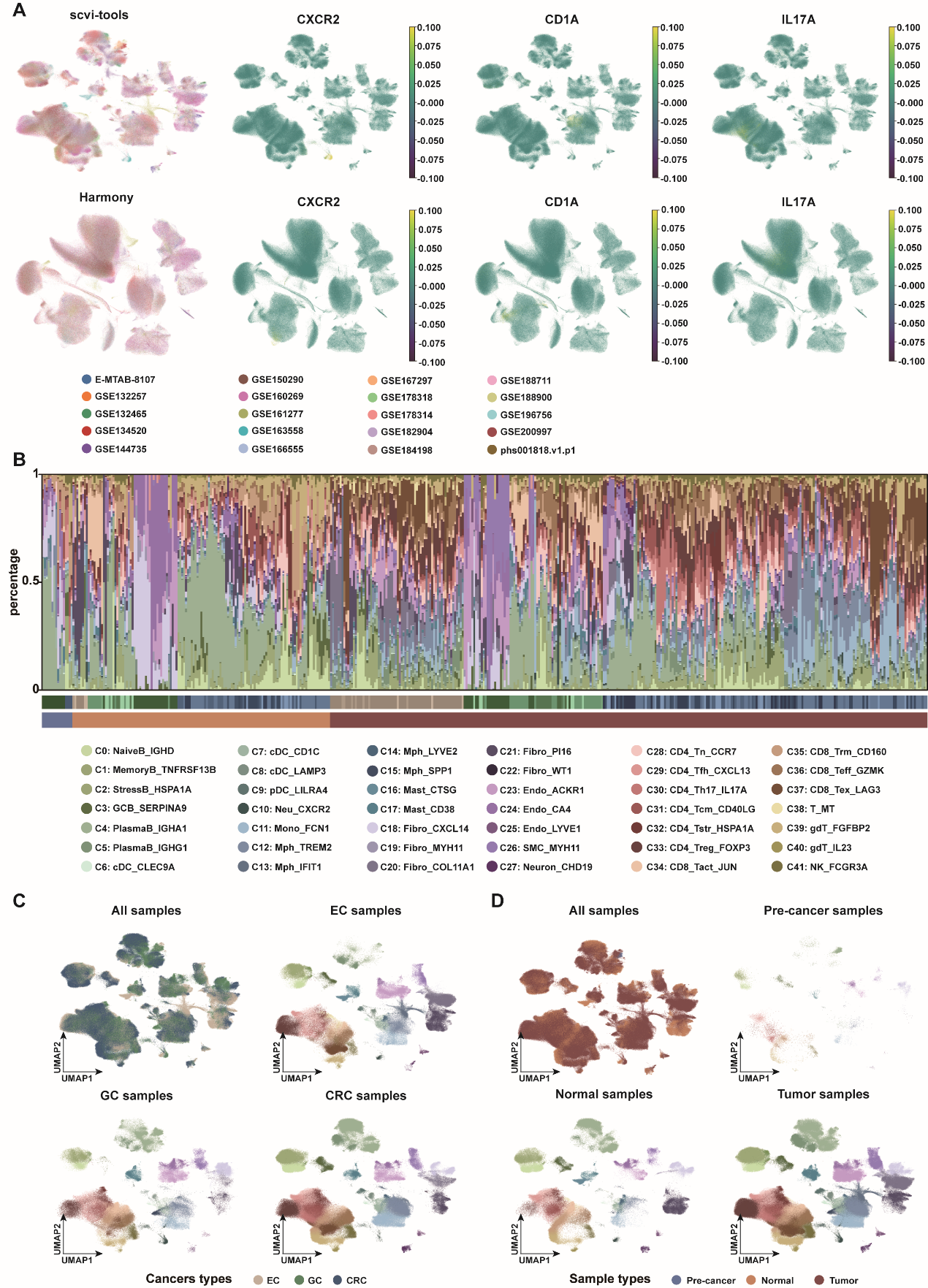


**Supplementary Figure S11. Related to Figure 4. (A).** UMAP visualization of unsupervised clustering and annotation of non-epithelial cells, integrated after batch effect correction using scvi-tools. From left to right, colored by datasets and showing the relative expression of CXCR2, CD1A, IL17A. **(B).** Stacked bar plot illustrating the proportion of cell subtypes in each sample, colored by cell subtypes. **(C)**. Top left: UMAP visualization of unsupervised clustering and annotation of non-epithelial cells, colored by cancer types. Top right: UMAP visualization of non-epithelial cells derived from esophageal cancer patients, colored by cell subtypes. Bottom left: UMAP visualization of non-epithelial cells derived from gastric cancer patients, colored by cell subtypes. Bottom right: UMAP visualization of non-epithelial cells derived from colorectal cancer patients, colored by cell subtypes. **(D)**. Top left: UMAP visualization of unsupervised clustering and annotation of non-epithelial cells, colored by sample types. Top right: UMAP visualization of non-epithelial cells derived from pre-cancer samples, colored by cell subtypes. Bottom left: UMAP visualization of non-epithelial cells derived from normal samples of patients, colored by cell subtypes. Bottom right: UMAP visualization of non-epithelial cells derived from tumor samples of patients, colored by cell subtypes.


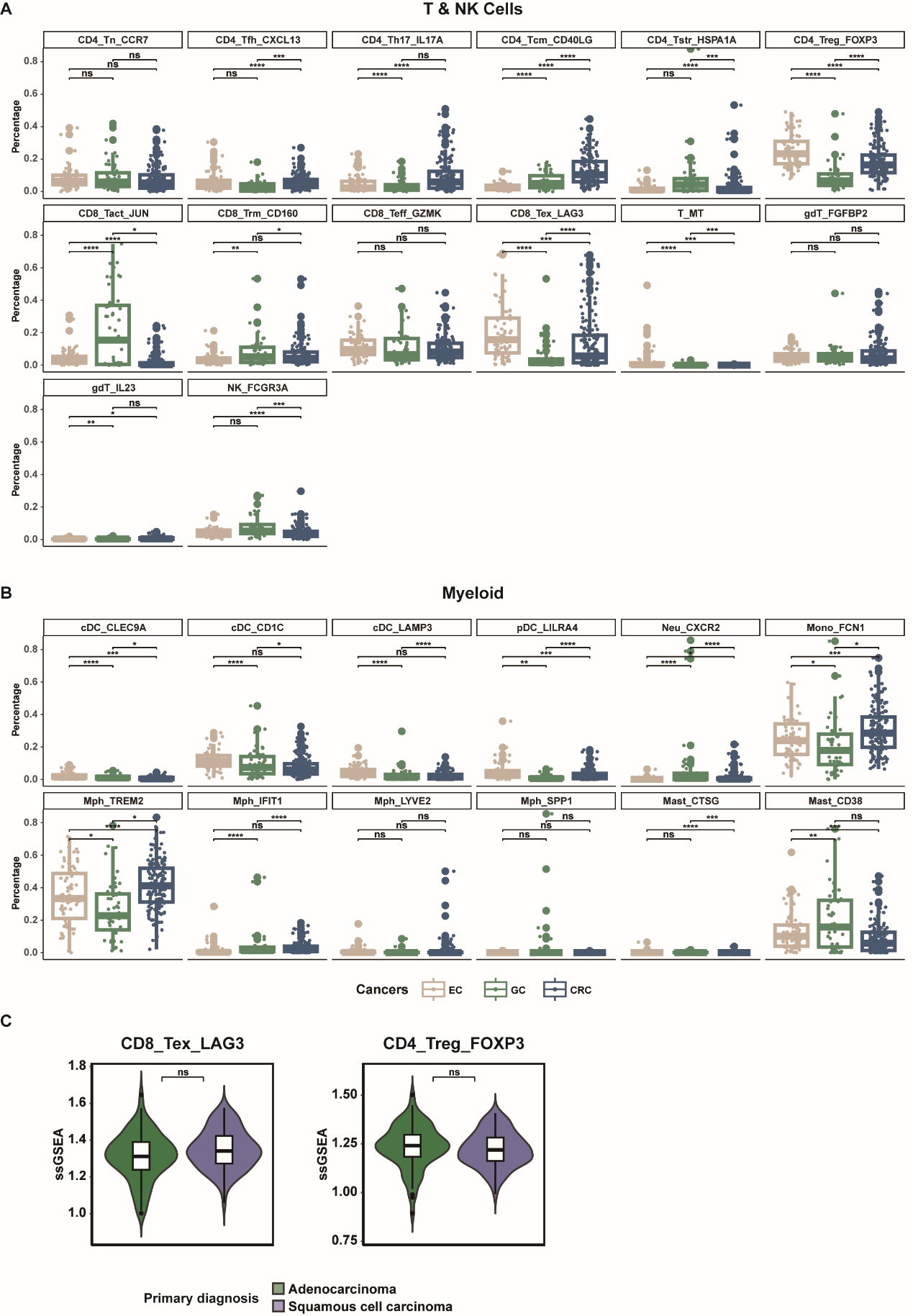


**Supplementary Figure S12. Related to Figure 4. (A).** Comparison of T and NK subtypes among cancer types, normalized by the total number of T and NK cells in each subtype. The number of samples from left to right: EC sample (n = 70), GC sample (n = 47), CRC sample (n = 155). (**B).** Comparison of Myeloid subtypes among cancer types, normalized by the total number of myeloid cells in each subtype. The number of samples from left to right: EC sample (n = 69), GC sample (n = 47), CRC sample (n = 150). **(C).** Violin plots showing the signature of immune cells between adenocarcinoma and squamous cell carcinoma in EC samples using TCGA data. Left: exhausted CD8 cells (CD8_Tex_LAG3). Right: Tregs (CD4_Treg_FOXP3). The number of samples in each group: adenocarcinoma (*n* = 87), squamous cell carcinoma (*n* = 89).

**
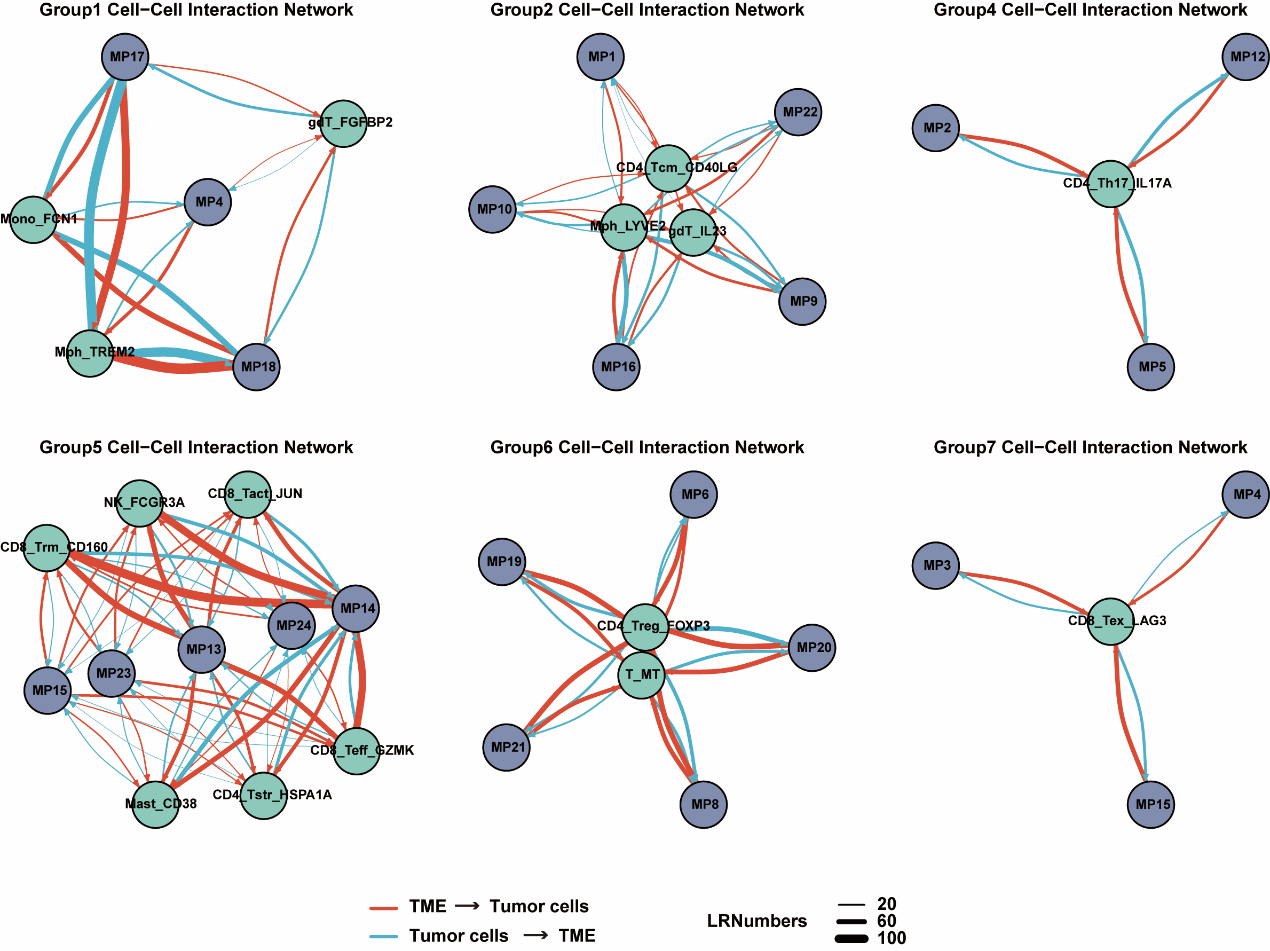
**

**Supplementary Figure S13. Related to** **Figure 5.** Network diagrams illustrating the tumor-TME interaction landscapes across six niches, with edge widths representing the number of ligand-receptor pairs per interaction type and colored by distinguishing interaction type.


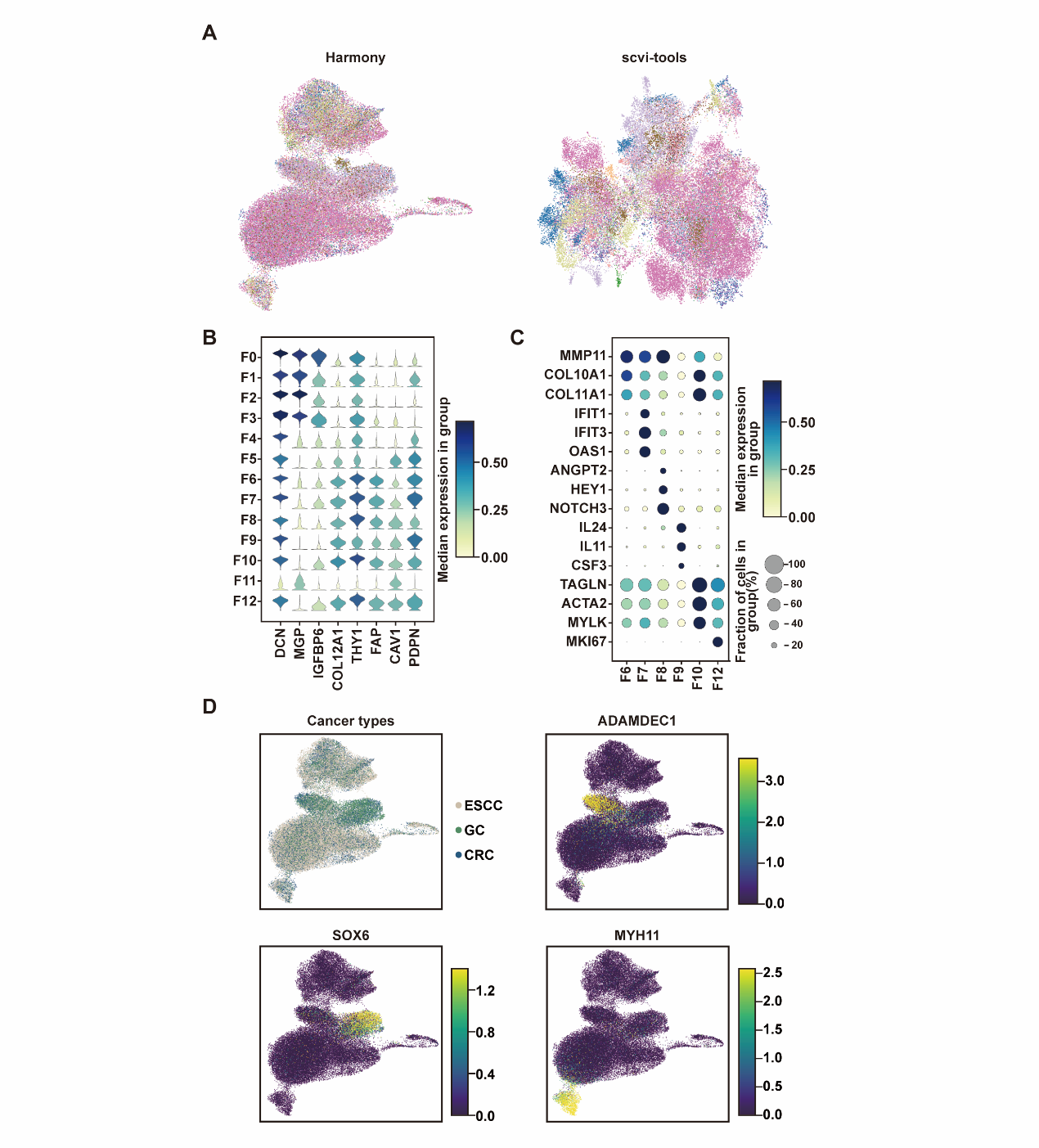


**Supplementary Figure S14. Related to Figure 6. (A).** UMAP visualization of unsupervised clustering and annotation of fibroblasts cells derived from tumor samples, colored by datasets. Left: batch effect correction using Harmony. Right: batch effect correction using scvi-tools. **(B).** Violin plots showing the expression of normal fibroblasts (NFs) and cancer-associated fibroblasts (CAFs) marker genes, colored by scaled relative genes expression. **(C).** Dot plot validation the function of CAFs clusters based on the proportions and scaled relative expression of functional genes. Colors represent scaled normalized gene expression, while dot size reflects the fraction of each gene in the corresponding cell type. **(D).** UMAP visualization of unsupervised clustering and annotation of fibroblast cells derived from tumor samples. From left and right, colored by cancer types, ADAM28, SOX6, and MYH11 relative expression.


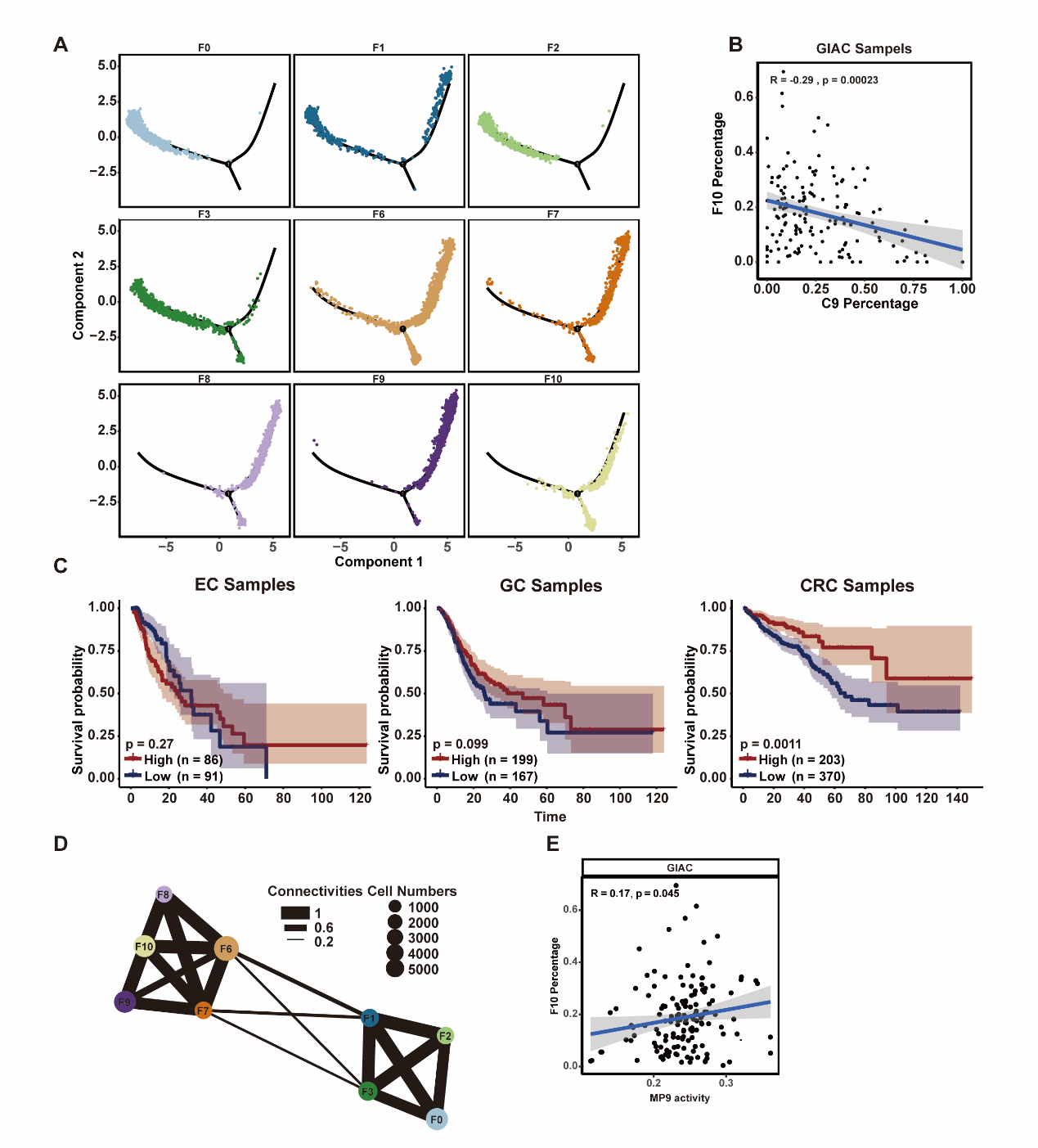


**Supplementary Figure S15. Related to Figure 6. (A).** Inferred developmental trajectory of fibroblasts, with facets representing different fibroblast clusters and colored according to their cluster assignments. **(B).** Scatter plot illustrating the correlation between C9 and C10 proportions across samples. Each point represents a sample, with the x-axis indicating the proportion of C9 and the y-axis showing the proportion of C10. Correlation strength is quantified by the Pearson correlation coefficient. The number of samples (n = 158). **(C).** Kaplan-Meier overall survival curves for TCGA patients stratified by the gene signatures of C9 and C10. Left: esophageal cancer patients, right: gastric cancer patients. The number of samples (n = 158). **(D).** The partition-based graph abstraction (PAGA) illustrating the relationship among fibroblasts clusters. The width of the edges represents the strength of the associations between clusters, while the size of the nodes corresponds to the number of cells in each cluster. Edges with connectively higher than 0.15 showed in the graph. **(E).** Scatter plot illustrating the correlation between MP9 activity and C10 proportions across samples. Each point represents a sample and correlation strength is quantified by the Pearson correlation coefficient. The number of samples (n = 152).


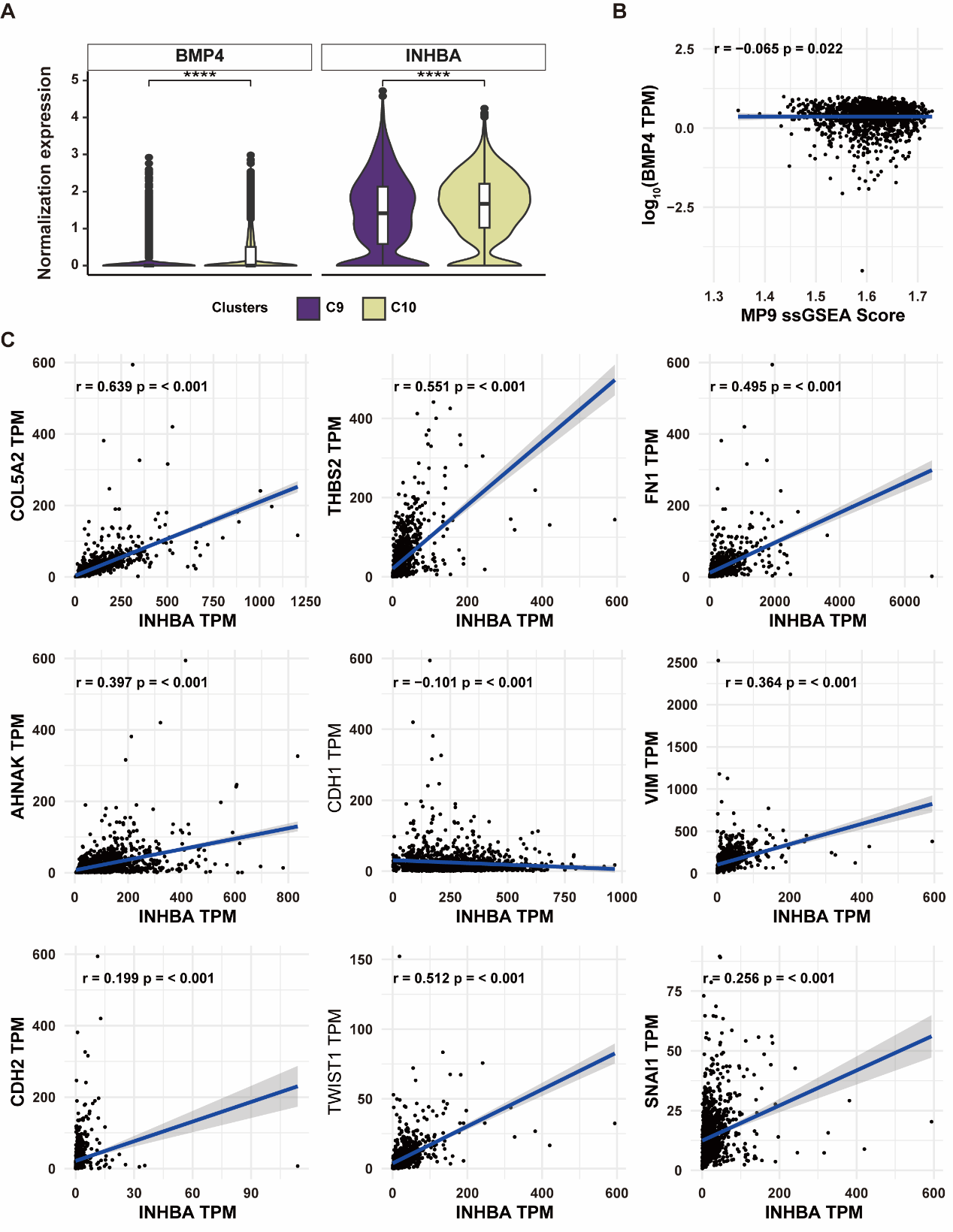


**Supplementary Figure S16. Related to Figure 6.** **(A).** Comparison of potential ligand expression between F9 and F10 fibroblasts. Violin plots showing the distribution of gene expression in the two groups. Left panel: BMP4 gene. Right panel: INHBA gene. **(B).** Correlation between log10(BMP4 TPM) and MP9 activity score across TCGA tumor samples. Each point represents an individual tumor sample (n=1233). The solid blue line indicates the linear regression fit, with the shaded area representing the 95% confidence interval. **(C).** Correlation analysis between INHBA TPM and the TPM levels of potential targets or EMT-related marker genes across TCGA tumor samples.


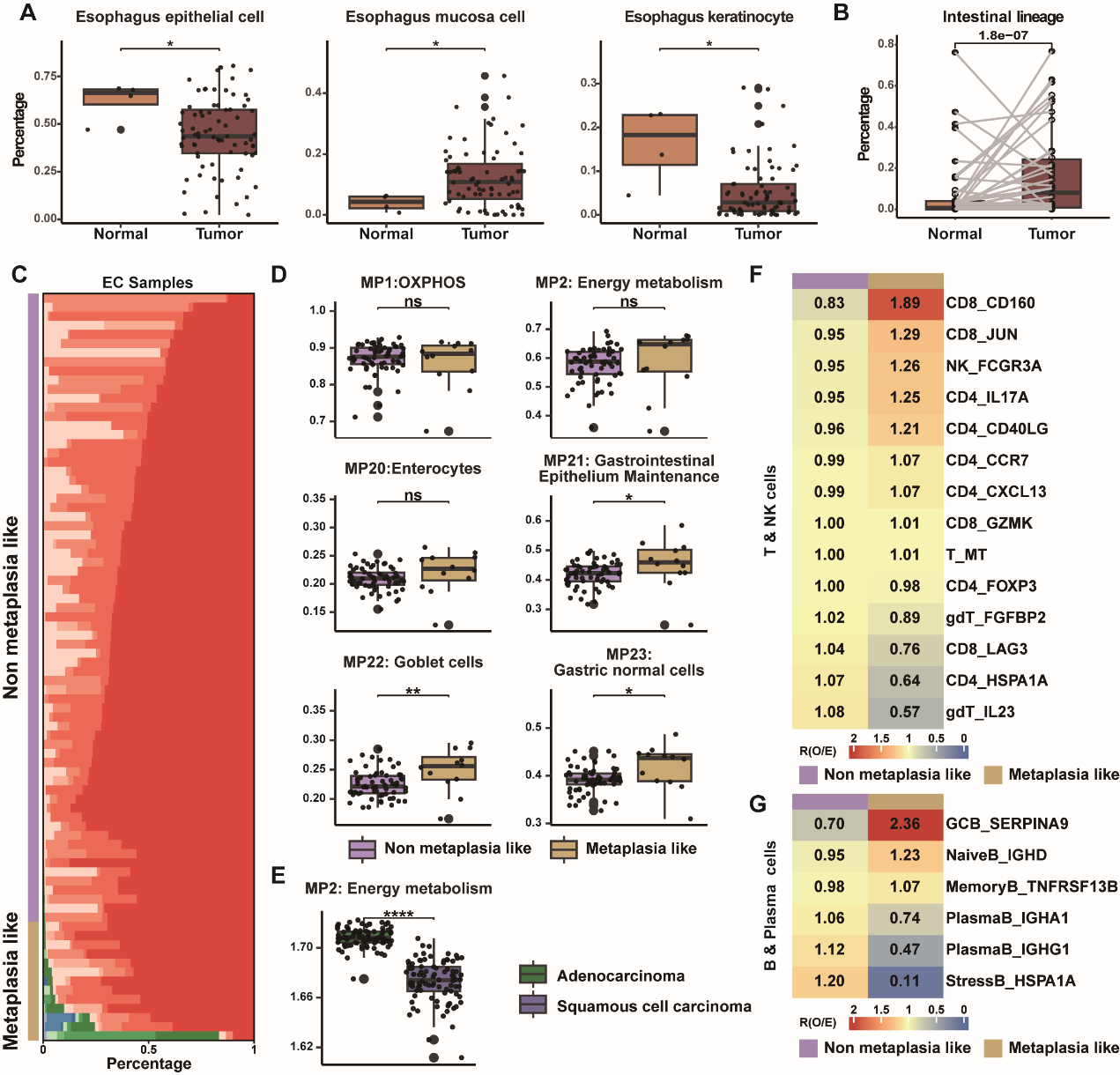


**Supplementary Figure S17. Related to Figure 7. (A).** Comparison of cell lineage proportions between normal and tumor samples in esophageal cancer. From left to right sequentially, esophageal epithelial cell, esophageal mucosa cell and esophageal keratinocyte. The number of samples: Normal samples (n = 4), Tumor samples (n = 75). **(B).** Comparison of intestinal lineage proportion between normal and tumor in paired samples from gastric cancer. The number of paired samples: Normal samples (n = 42), Tumor sample (n = 42). **(C).** Stacked bar plots illustrating the proportions of cell lineages in each esophageal cancer sample. Left annotation indicates the classification groups based on the proportion of metaplastic cell lineage including gastric and colorectal cell lineage. The number of samples in each group: metaplastic like (n = 13), non-metaplastic like (n = 69). **(D).** Comparison of MP activity between gastric and intestinal groups in tumor samples from esophageal cancer. Top left: MP1 (OXPHOS). Top right: MP2 (Energy metabolism).

Middle left: MP20 (Enterocytes). Middle right: MP21 (Gastrointestinal epithelium maintenance). Bottom left: MP22 (Goblet cells). Bottom right: MP23 (Gastric normal cells). The number of samples in each group: non-metaplastic group sample (n = 63), metaplastic group sample (n = 12). **(E).** Comparison of MP2 (Energy metabolism) activity between adenocarcinoma and Squamous cell carcinoma in tumor samples from esophageal tumor samples in TCGA. The number of samples in each group: adenocarcinoma (*n* = 87), squamous cell carcinoma (*n* = 89). **(F).** Prevalence of T & NK clusters between metaplastic group and non-metaplastic group in esophageal tumor samples, the color of heatmap representing the value of Ro/e of each cell types in each cancer type. **(G).** Prevalence of B & Plasma clusters between metaplastic group and non-metaplastic group in esophageal tumor samples, the color of heatmap representing the value of Ro/e of each cell types in each cancer type.


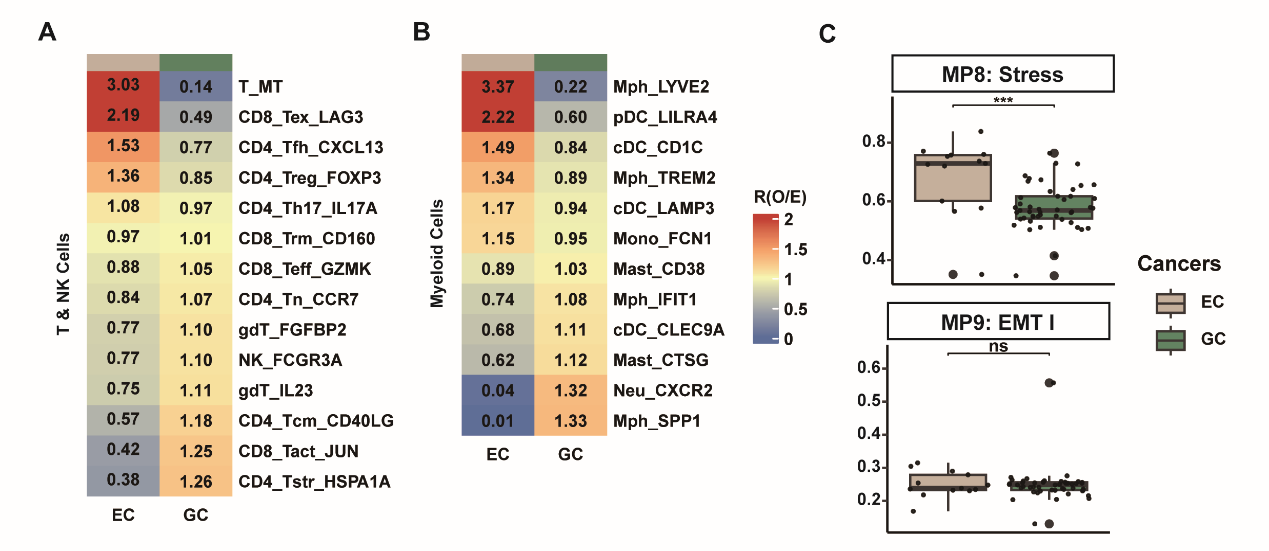


**Supplementary Figure S18. Related to Figure 7.** **(A).** Prevalence of T & NK clusters between metaplastic samples from EC and GC, the color of heatmap representing the value of Ro/e of each cell type in each cancer type. **(B).** Prevalence of B & Plasma clusters between metaplastic samples from EC and GC, the color of heatmap representing the value of Ro/e of each cell type in each cancer type. **(C).** Comparison of MPs activity between metaplastic samples from EC and GC. Left: MP8 (Stress). Right: MP8 (Stress). Left: MP8 (EMT I).
